# Revolutionary Point‐of‐Care Wearable Diagnostics for Early Disease Detection and Biomarker Discovery through Intelligent Technologies

**DOI:** 10.1002/advs.202400595

**Published:** 2024-07-03

**Authors:** Fatemeh Haghayegh, Alireza Norouziazad, Elnaz Haghani, Ariel Avraham Feygin, Reza Hamed Rahimi, Hamidreza Akbari Ghavamabadi, Deniz Sadighbayan, Faress Madhoun, Manos Papagelis, Tina Felfeli, Razieh Salahandish

**Affiliations:** ^1^ Laboratory of Advanced Biotechnologies for Health Assessments (Lab‐HA) Biomedical Engineering Program Lassonde School of Engineering York University Toronto M3J 1P3 Canada; ^2^ Department of Electrical Engineering and Computer Science (EECS) Lassonde School of Engineering York University Toronto ON M3J 1P3 Canada; ^3^ Department of Biology Faculty of Science York University Toronto ON M3J 1P3 Canada; ^4^ Department of Ophthalmology and Vision Sciences University of Toronto Ontario M5T 3A9 Canada; ^5^ Institute of Health Policy Management and Evaluation University of Toronto Ontario M5T 3M6 Canada

**Keywords:** AI‐assisted biomarker discovery, high‐throughput omics and clinical study, intelligent diagnostics, point‐of‐care biomarker detection platforms, precision medicine, remote disease detection, wearable health monitoring

## Abstract

Early‐stage disease detection, particularly in **P**oint‐**O**f‐**C**are (**POC**) wearable formats, assumes pivotal role in advancing healthcare services and precision‐medicine. Public benefits of early detection extend beyond cost‐effectively promoting healthcare outcomes, to also include reducing the risk of comorbid diseases. Technological advancements enabling POC biomarker recognition empower discovery of new markers for various health conditions. Integration of POC wearables for biomarker detection with intelligent frameworks represents ground‐breaking innovations enabling automation of operations, conducting advanced large‐scale data analysis, generating predictive models, and facilitating remote and guided clinical decision‐making. These advancements substantially alleviate socioeconomic burdens, creating a paradigm shift in diagnostics, and revolutionizing medical assessments and technology development. This review explores critical topics and recent progress in development of 1) POC systems and wearable solutions for early disease detection and physiological monitoring, as well as 2) discussing current trends in adoption of smart technologies within clinical settings and in developing biological assays, and ultimately 3) exploring utilities of POC systems and smart platforms for biomarker discovery. Additionally, the review explores technology translation from research labs to broader applications. It also addresses associated risks, biases, and challenges of widespread **A**rtificial **I**ntelligence (**AI**) integration in diagnostics systems, while systematically outlining potential prospects, current challenges, and opportunities.

## Graphical abstract

Point‐of‐care systems, particularly in their wearable format, play a pivotal role in transforming the diagnostics and healthcare realm. Integration of these high‐tech platforms with intelligent systems, such as artificial intelligence (AI), is a promising path for revolutionizing early disease detection, in vitro bioassays, medical image/data analysis, and conducting clinical studies for introducing novel disease biomarkers.

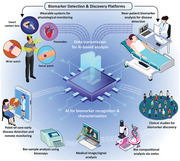

## 1 Introduction

Within any healthcare system, timely and accurate diagnosis of health conditions assumes a pivotal role in effective disease management, monitoring of disease progression, and alleviating the financial, psychological, and social strains of disease.^[^ ^1^ ^]^ Early detection of health conditions is hence imperative as it greatly influences the selected treatment plan, and subsequently enhances health outcomes.^[^ ^2^ ^]^ A vast majority of recent advancements in the realm of precision medicine, advanced diagnostics, and enhanced medical interventions, which have led to a significant increase in survival rates and prolonged life expectancy in the last decades, are mainly pertinent to the detection of disease‐specific “biomarkers”.^[^ ^3^ ^]^ Biomarkers are the biological attributes primarily linked to biomolecules, imaging features, or biophysical parameters, that are associated with a particular disease, or an indicator of biological processes.^[^ ^4^ ^]^ By employing a combination of such biologically plausible manifestations, healthcare practitioners can now more accurately diagnose diseases, and provide more effective treatment plans and therapeutic interventions.^[^ ^3^, ^5^ ^]^ Thus, the characteristics and features of testing methods and devices employed for the detection of various biomarkers, such as biosample compositional analysis or protocols for the identification of anomalies in medical images/signals, are of paramount importance in the diagnosis procedure.^[^ ^6^ ^]^ The convergence of technological breakthroughs in engineering, biology, chemistry, and medicine has transformed the field of disease diagnosis over the past decades through the invention of state‐of‐the‐art devices and laboratory techniques, that facilitate more reliable and precise biomarker detection than ever before.^[^ ^7^ ^]^ It is apparent that near‐patient diagnostic solutions such as **P**oint‐**O**f‐**C**are (**POC**) platforms, wearable systems,^[^ ^8^ ^]^ and intelligent technologies, mainly operated by **A**rtificial **I**ntelligence (**AI**) algorithms, are still finding their place in real‐world clinical settings as standard or complimentary disease detection techniques. Despite being at an early stage in their practical clinical application, these innovations hold substantial promise in redefining standard clinical practices for early disease management.^[^ ^9^ ^]^

While highly advanced tools and solutions are vital for proper diagnosis, the biological moieties introduced as the disease bioindicators shall also be physiologically pertinent. Large‐scale clinical studies are requisite to corroborate the role of these markers in disease pathogenesis, as well as depict meaningful attributions to the pathophysiological states of the diseases and disorders in focus compared to healthy conditions.^[^ ^10^ ^]^ Conventionally, case‐control studies, involving patient and healthy cohorts recruitment and the analysis of bio‐compositional variations in clinical samples or examination of medical images/signals data, are being conducted to determine the disease‐specific biomarkers.^[^ ^11^ ^]^ In the clinical context, the identified biomarkers not only contribute to disease detection or prevention but also play an important role in monitoring the drug response in the field of pharmacoproteomics for prevalent diseases such as cancers.^[^ ^12^ ^]^ Particularly for lethal health conditions, such as cardiovascular diseases, biomarker discovery is significantly important as it can greatly contribute to foreseeing and preventing morbidities and mortalities.^[^ ^13^ ^]^ In this realm, these efforts, which range from transcriptome profiling to proteomics and metabolomics shed light on the molecular mechanisms of the diseases or biological pathways, and also genomic markers that might be involved in drug resistance or sensitivity.^[^ ^14^ ^]^ The advancements in high‐throughput technologies and methodologies for accurate bio‐compositional analysis, such as mass‐spectroscopy‐based protein and metabolite profiling, warrant attention. These techniques have proven effective not only in discovering novel indicators of well‐defined pathologies like cancer^[^ ^15^ ^]^ but also in the nuanced characterization of clinical conditions that have long been challenging to precisely identify, such as various psychiatric conditions.^[^ ^16^ ^]^

POC solutions and wearable platforms offer a unique system through which the biomarkers can be monitored remotely and longitudinally. In any format, they provide this opportunity for individuals to potentially assess their health condition without the need for regular visits for frequent testing and physician interventions.^[^ ^17^ ^]^ The automated nature of these platforms empowers biomarker detection for the general population with no specific training in lab skills.^[^ ^18^ ^]^ Also, such systems offer the capabilities needed for the detection of multiple markers in one single assay run. Physicians and clinicians are also interested in investigating diseases through a “panel‐based” strategy, i.e., via multiple markers.^[^ ^19^ ^]^ In clinical terms, having multiple indicators of a disease, e.g. different proteins or additional disease‐specific imaging/signal features, can assist with more reliable diagnosis, compared to only relying on one biomarker for disease detection. This mitigates the risk of misdiagnosis arising from the limited specificity of certain markers, or the circumstance wherein certain biomolecules might serve as characteristic indicators for multiple health conditions or disorders. Microfluidics‐integrated wearable systems, and other types of POC platforms, leverage their miniaturized nature, to offer multiplexing features.^[^ ^20^ ^]^ Some of the current challenges in the field of diagnostics and biomarker discovery still include the invasiveness of sample collection procedures, the lack of standardized protocols, and the accuracy and expansiveness of the bioassays used for chemical analysis. Some of these challenges have been aimed to rectify using novel technologies such as POC platforms and wearable smart systems.^[^ ^21^ ^]^ Beyond their diagnostic value, these systems offer unique characteristics that enable in situ detection of a panel of biomarkers, which contributes to testing larger cohorts, resulting in the identification of reliable disease markers. Furthermore, miniaturized platforms such as POC systems or wearable patches considerably reduce the financial implications of clinical studies in biomarker discovery. These advancements reduce the necessity for recurrent patient visits for sample procurement and centralized laboratory testing while empowering wireless and remote practice of personalized medicine.

With the swift advancement of AI tools, in particular, the integration of smart systems and machine learning algorithms into diagnostics solutions, a new era of innovation has been inaugurated in the realm of intelligent biomarker detection systems. The AI algorithms have been developed to assist clinical decision‐making through automated labeling and analyses of the medical images and signals (digital pathology), as well as molecular pathology in routine laboratory testing.^[^ ^22^ ^]^ Pertaining to the identification of biomarkers for disease detection, AI tools can be particularly useful as they can analyze large sets of data and extract patterns that are normally overlooked by human professionals. These data analytic approaches have been exercised in various omics disciplines,^[^ ^23^ ^]^ such as proteomics, and metabolomics, for instance in the realm of precision oncology^[^ ^24^ ^]^ or in genomics.^[^ ^25^ ^]^ While the benefits of incorporating smart algorithms are evident, there are still challenges associated with their integration into biomarker discovery practice, which includes overfitting, or discovery of false associations^[^ ^26^ ^]^ which leads to misdiagnosis. Consequently, intensified efforts are needed in this domain, for instance in developing unbiased algorithms,^[^ ^27^ ^]^ for the AI‐assisted discovery of robust disease‐specific biomarkers. The potential role of AI‐assisted methodologies in designing POC systems, analyzing the patterns of detected biomarkers, and predicting the outcomes of interventions, can transform the status quo on near‐patient and personalized medicine applications. A pertinent illustration of such endeavors is the current utilization of various **D**eep **L**earning (**DL**) algorithms for the analysis of physiological parameters through novel wearable systems designed to monitor panic attacks.^[^ ^28^ ^]^

**Figure 1:**
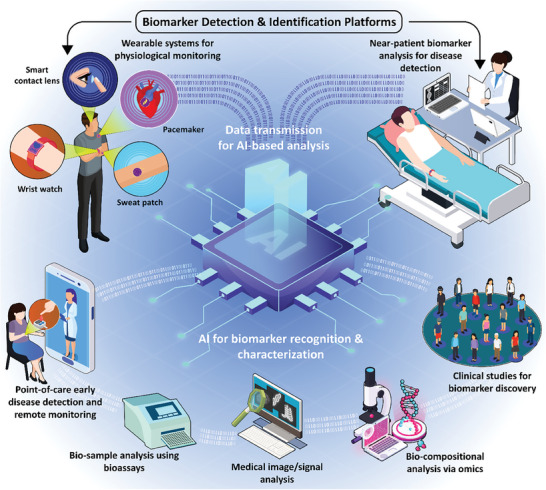
Schematic representation of the biomarker detection and identification platforms. Systems for the detection of various biomarkers are employed for disease diagnosis in a near‐patient format, in the presence of patients in clinical settings, or by other approaches such as physiological monitoring through wearable systems. Wearable platforms, such as smart contact lenses, pacemakers, sweat patches, and digital wristwatches provide invaluable insight into the physiological conditions and individual's health status. Biomarker detection and identification platforms for bedside, or near‐patient, applications are utilized for assessing the biocomposition of the patient biosamples and detecting specific biomarkers, or for medical imaging or medical signal analysis. Integrated with Artificial Intelligence (AI), upon transmission of data, preferably wirelessly, these systems can be used for a variety of purposes in the realm of biomarker recognition and characterization. This includes early detection of disease and Point‐Of‐Care (POC) applications for remote assessment of the individual's health status, with the testing performed by the patients outside clinical settings, conducting biological assays for biomolecule detection and biosample analysis, automated AI‐based image/signal analysis, omics for bio‐compositional analysis of biospecimens, and in the area of conducting clinical studies for biomarker discovery.

In this review paper, we intend to explore the most recent advancements in 1) POC and wearable systems that enable the detection of disease‐specific indicators for early disease monitoring. Our analysis extends to evaluating the applications of the 2) computational methods, intelligent systems and algorithms, and interactive tools in clinical settings, overviewing the tailored use of these techniques in medical image/signal analysis, biological assays, drug discovery, and laboratory visualization. Additionally, we aim to examine the 3) applications of such POC and wearable systems in biomarker discovery, as well as the necessity and clinical significance of integrating smart solutions and AI‐based analysis into near‐patient biomarker detection practices. **Figure** **1** illustrates a schematic overview of the subjects addressed in this review. The path toward receiving health authority approvals and commercializing the technologies developed for AI‐based disease tracking and biomarker discovery will also be discussed. This discussion will extend to providing an outlook into the significance of these innovations for health industry partners, associated risks and errors encountered in the translational efforts in this area, as well as prospective opportunities that lie ahead.

## 2 POC Systems and Wearable Solutions

### 2.1 Technological Advances in In Vitro Diagnostic Development

**I**n **V**itro **D**iagnostic (**IVD**) devices are prominent technologies used in disease detection.^[^ ^29^ ^]^ For successful early‐stage diagnosis and precise subtyping of diseases, two crucial factors need to be addressed in IVD systems. These include the selection of appropriate targets that provide timely feedback and the design of high‐performance device integration.^[^ ^30^ ^]^ POC solutions have emerged for enhancing the applicability of IVDs, outside of centralized laboratories, toward accessible and affordable systems that can be operated feasibly by the patients or in the “near‐patient” format. By offering diagnostic results with minimal delay, POC diagnostics enable healthcare providers to make timely treatment decisions and provide immediate guidance to patients,^[^ ^31^ ^]^ mostly without requiring any special equipment or training.^[^ ^32^ ^]^ POC Testing (POCT) has garnered significant interest due to its portability and user‐friendly interface, enabling rapid and precise test results directly at the sampling location. A diverse array of technologies has been employed in the POC systems including microfluidics.^[^ ^33^ ^]^ This enables the miniaturization of working units, allowing for the handling of small sample volumes and enhanced integration capabilities. This, in turn, supports multiplexed testing.^[^ ^34^ ^]^ Additionally, various forms of Micro‐Electromechanical Systems (MEMS) have also been integrated into these platforms.^[^ ^35^ ^]^ These technologies are amalgamated with biomolecule detection strategies, mainly in a miniaturized format, and are further augmented by nanostructures. These combinations empower on‐site recognition of disease‐associated biomarkers.

**Figure 2:**
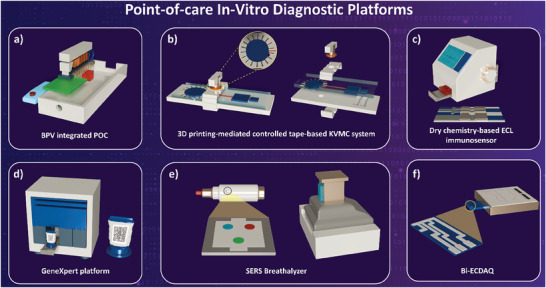
Examples of POC Testing (POCT) solutions for biomarker detection and In Vitro Diagnostics (IVDs). a) A POC detection platform for the detection of severe acute respiratory syndrome coronavirus 2 (SARS‐CoV‐2) spike protein based on a self‐assembled plasmonic nanoprobe array mechanism enabled through Bioinspired Plasmo‐Virus (BPV) particle synthesis, Reproduced with permission.^[^ ^40^ ^]^ Copyright 2023, American Chemical Society. b) Microfluidic system with integrated tapes for multiplexed detection of inflammatory markers of C‐reactive protein (CRP), Procalcitonin (PCT), and Interleukin‐6 (IL‐6) for early clinical diagnosis of sepsis, Reproduced with permission.^[^ ^42^ ^]^ Copyright 2022, Elsevier. c) A dry chemistry‐based bipolar Electrochemiluminescence (ECL) immunoassay system for POCT of Alzheimer‐associated neuronal thread protein (AD7c‐NTP), Reproduced with permission.^[^ ^47^ ^]^ Copyright 2023, American Chemical Society. d) GeneXpert platform, a PCR‐based system for the detection of multiple diseases in various testing settings (Image Reproduced with permission from Cepheid),^[^ ^44^ ^]^ e) Mass screening of COVID‐19 via detection of breath volatile compounds using a SERS‐based breathalyzer, Reproduced with permission.^[^ ^45^ ^]^ Copyright 2022, American Chemical Society. f) POC detection of COVID‐19 using electrochemical dual immunosensing of the nucleocapsid protein (N‐protein) via electrochemical impedance spectroscopy (Bi‐ECDAQ), Reproduced with permission.^[^ ^58^ ^]^ Copyright 2022, Elsevier.

The conventional biomolecule detection strategies embedded into the IVDs encompass methods such as electrochemical sensors, and optical approaches including Surface‐Enhanced Raman Scattering (SERS) or Enzyme‐Linked Immunosorbent Assay (ELISA), in particular, in its digital format, along with colorimetric techniques.^[^ ^36^ ^]^ These techniques can be integrated into compact, on‐site systems for sample preparation and detection, including various forms of microfluidic system configurations. The sought‐after miniaturization is often realized by incorporating microfluidic paper‐based analysis devices (μPads)^[^ ^37^ ^]^ into biomarker detection solutions. These systems are enhanced using specific nanomaterials, functional layers and materials, and advanced sensors that eventually lead to the system's overall lower Limit of Detection (LODs), reduced susceptibility to contamination, and increased mechanical strength.^[^ ^38^ ^]^ **Table** **1** explores various POCT platforms developed in recent years for biomarker detection, discussing the target disease, the specification of the system in terms of sensitivity and figures of merit, as well as the sensing technology incorporated. A number of these platforms have also been schematically represented in **Figure** **2**.

**Table 1:** In vitro diagnostics and POC solutions.

Platform	Sensing Technology	Biospecimens	Sensitivity‐LOD‐LOQ‐Linear range	Disease	Biomarker	Clinical Samples	References
CAAB	PEC	Blood	Linear Response: 0.02 – 40 ng mL^−1^	Cancer	PSA	None	[39]
Hand‐Held POC Biosensor Incorporating the Plasmo‐Virus Particle‐Based Assay	LSPR NS	Fluid in the Upper Respiratory Tract	LOD: 14 pfu mL^−1^	COVID‐19	SARS‐CoV‐2	None	[40]
Immunosensor Based on an IMA	Impedimetric	Blood	LOD: 0.4 BAU mL^−1^	COVID‐19	SARS‐CoV‐2 Antibodies (IgG, IgM and IgA)	None	[41]
3D Printing‐Mediated Controlled Tape‐Based KVMC Tunable Microfluidics	CL	Blood	LOD: 0.23 µg mL^−1^, LOD: 0.14 ng mL^−1^, and LOD: 12.53 pg mL^−1^	Sepsis	CRP, PCT, and IL‐6	Yes 25 Patients, 25 Healthy Controls	[42]
ELISA Kit and Paper‐Based LFA Designed for Use Alongside Smartphones and Tablets	ELISA + Visual Detection on a LFA	Fluid in the Vaginal Canal	LOD (ng mL^−1^): 24.58 ± 11.93, LOQ (ng mL^−1^): 189.22 ± 184.98	Bacterial Vaginosis	VLY	Yes 27 Subjects	[43]
GeneXpert Platform	Liquid‐Based Cytology + Microscopic Examination	Mid‐Cavity Vaginal Fluid	Sensitivity: 91·7%	HPV – Cervical Cancer	HPV DNA	Yes 4285 Women	[44]
Hand‐Held SERS‐Based Breathalyzer	SERS	Breath	Sensitivity: >95%	COVID‐19	BVOCs such as Aldehydes, Ketones, and Alcohols	Yes 501 Subjects	[45]
Enzyme‐Based Color Bar‐Style LFS for Equipment‐Free Semi‐Quantitative Determination of UO	Colorimetric in a LFA	Urine	LOD: Less than 0.3 mm	Urolithiasis	Calcium Oxalate	None	[46]
Dry Chemistry‐Based Bipolar ECL Immunoassay Device	ECL	Urine	LOD: 0.15 pg mL^−1^	AD	AD7c‐NTP	Yes Samples of 5 Healthy Adult Women	[47]
Multimodal Capture Independent LFIA Biosensor Based on AuNF‐PMBA NMs	Colorimetric, SERS, and Photothermal	Urine	LOD: 10^3^ cfu mL^−1^, LOD: 10^2^ cfu mL^−1^, LOD: 10^2^ cfu mL^−1^	UTI	E. coli	Yes 15 Subjects	[48]
LFA Aptasensor	LFA	Sweat	LOD: 1 ng mL^−1^	Stress	Cortisol	None	[49]
SERS‐Based Sandwich Immunoassays for Multiplexed Detection	LFA + SERS	Blood	LOD: 0.72 ng mL^−1^ and LOD: 7.67 ng mL^−1^	Zika, Dengue	ZIKV NS1 and DENV NS1	None	[50]
Smartphone‐Based Diagnostic Platform	RT‐LAMP	Urine, Saliva, and Blood	LOD_95_ (ZINKV): 22 PFU mL^−1^, LOD_50_((ZINKV): 4.9 PFU mL^−1^	Zika, Chikungunya, and Dengue viruses	ZIKV, CHIKV, and DENV	None	[51]
LAMP Coupled with NP‐Based Biosensor	LAMP + LFB	NPS samples	Sensitivity: 50 fg per reaction	MP	CARDS Toxin Gene	Yes 100 Children	[52]
Rapid Isothermal Amplification and Portable Detection System	RT‐LAMP	NPS samples	LOD: 50 RNA copies per µL	COVID‐19	SARS‐CoV‐2	Yes 20 Subjects	[53]
Rapid and Fully Microfluidic Ebola Virus Detection with CRISPRCas13a	CRISPR	Blood	LOD: 20 pfu/mL (5.45 × 10^7^ copies mL^−1^) of purified Ebola RNA	Ebola	Ebola RNA	None	[54]
A POC Test Comprising an IC Strip and a Smartphone Reader	LFA	Blood	LOD: 200 ng ml^−1^	Ebola	EVD IgG	Yes 121 Serum Samples: 90 from Survivors and 31 from Noninfected Controls	[55]
Alere q HIV‐1/2 Detect Test	NAT‐Based Tests (PCR)	Blood	300 copies mL^−1^ or 8 copies in 25 µL	HIV‐1/2	HIV RNA	Yes 13 Subjects	[56]
Rapid Paper‐Based Colorimetric Detection	Enzyme‐Based Colorimetric	ISF	Linear range: 0 – 10 mm	Diabetes	Glucose	None	[57]
Bi‐ECDAQ	EIS	Spiked samples and clinical NSP samples	WE1, LOD: 116 fg mL^−1^, WE2, LOD: 150 fg mL^−1^	COVID‐19	SARS‐CoV‐2 N‐Protein	Yes 7 SARS‐CoV‐2 Positive 7 Negative Controls	[58]
BiSense	EIS	Spiked samples and clinical NSP samples	WE1, LOD: 56 fg mL^−1^, WE2, LOD: 68 fg mL^−1^	COVID‐19	SARS‐CoV‐2 N‐Protein	22 Subjects	[59]

AD7c‐NTP: Alzheimer‐associated neuronal thread protein, AD: Alzheimer disease, AuNF: Au nanoflower, BVOCs: breath volatile organic compounds, CAAB: portable cancer‐detection antigen assay box, CARDS: community‐acquired respiratory distress syndrome, CHIKV: Chikungunya virus, CL: Chemiluminescent CRISPRCas13a: clustered regularly interspaced short palindromic repeats and CRISPR‐associated protein 13a, CRP: C‐reactive protein, DENV NS1: Dengue Virus nonstructural protein 1, ECL: electrochemiluminescence, E. coli: escherichia coli, EIS: electrochemical impedance spectroscopy, ELISA: enzyme‐linked immunosorbent assay, EVD: ebola virus disease, HIV‐1/2: human immunodeficiency Virus types 1 and 2, HPV: human Papillomavirus, IC: Immunochromatographic, IgA: immunoglobulin A, IgG: immunoglobulin G, IgM: immunoglobulin M, IL‐6: interleukin‐6, IMA: Interdigitated Microelectrode Array, ISF: interstitial fluid, KVMC: key valve microfluidic chip, LFA: lateral flow assay, LFB: lateral flow biosensor, LFIA: lateral flow immunoassay, LFS: lateral flow strip, LSPR: localized surface plasmon resonance, MP: mycoplasma pneumonia, NAT: nucleic acid test, NMs: nanomaterials, N‐protein: nucleocapsid protein, NP: nanoparticle, NPS: nasopharyngeal swab, NS: nanostructures, PCR: polymerase chain reaction, PCT: procalcitonin, PEC: photoelectrochemical, PMBA: p‐mercaptophenylboronic acid, POC: point‐of‐care, PSA: prostate‐specific antigen, RT‐LAMP: reverse‐transcription loop‐mediated isothermal amplification, SARS‐CoV‐2: severe acute respiratory syndrome coronavirus 2, SERS: surface‐enhanced raman scattering, UO: urinary oxalate, UTI: urinary tract infections, VLY: vaginolysin, ZIKV NS1: zika virus nanostructural protein 1.

**Figure 3:**
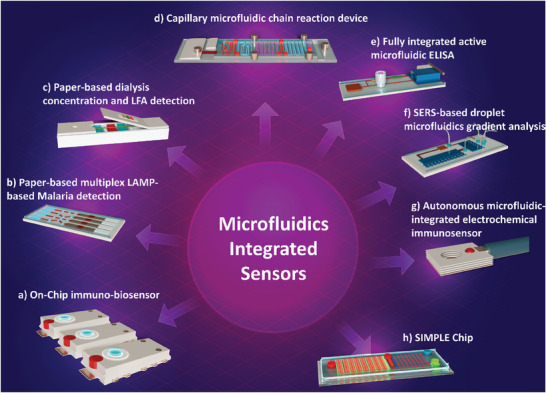
Microfluidics integrated biosensors. a) Immunobiosensor on a chip for electrochemical detection of COVID‐19 in a self‐powered microfluidic system, Reproduced with permission.^[^ ^63^ ^]^ Copyright 2022, Royal Society of Chemistry. b) Multiplexed DNA detection for Malaria diagnosis using an origami‐based paper microfluidic system and lateral flow detection, Reproduced with permission.^[^ ^64^ ^]^ Copyright 2019, PNAS. c) Paper‐based sample concentration technique in the Lateral Flow Assay (LFA) for detection of Human Immunodeficiency Virus (HIV) nucleic acid, Reproduced with permission.^[^ ^65^ ^]^ Copyright 2016, Elsevier. d) Programmed passive microfluidic capillary system and embedded Enzyme‐Linked Immunosorbent Assay (ELISA) for detection of SARS‐CoV‐2 antibodies, Reproduced with permission.^[^ ^66^ ^]^ Copyright 2022, Springer Nature. e) Active microfluidic system for rapid detection of cardiac troponin I (cTnI) through on‐chip ELISA, Reproduced with permission.^[^ ^67^ ^]^ Copyright 2021, Springer Nature. f) Droplet microfluidic system and integrated Surface‐Enhanced Raman spectroscopy (SERS)‐based sensor, Reproduced with permission.^[^ ^68^ ^]^ Copyright 2019, Royal Society of Chemistry. g) Autonomous microfluidic system coupled with electrochemical immunosensor for detection of Glial Fibrillary Acidic Proteins (GFAP), Reproduced with permission.^[^ ^69^ ^]^ Copyright 2022, Royal Society of Chemistry. h) Detection of Staphylococcus aureus DNA via a low‐cost microfluidic integrated isothermal amplification and on‐site nucleic acid quantification; (SIMPLE: Self‐powered Integrated Microfluidic Point‐of‐care Low‐cost Enabling) chip, Reproduced with permission.^[^ ^70^ ^]^ Copyright 2017, Science.

Integration of advanced sensors with microfluidic components will also result in precise fluid control, reduced reagent consumption, and feasible multiplexed sensing,^[^ ^60^ ^]^ as illustrated by several of the most advanced systems innovated in recent years in **Figure** **3**. With all such advancements, the IVD industry plays a pivotal role in translating research into practice. Historically, the methylation biomarker detection sector struggled to generate significant returns on investments and faced regulatory challenges in the IVD market. However, recent breakthroughs by entities such as EXACT Sciences Corporation, a molecular diagnostic company that has made significant strides in colorectal cancer screening with a test incorporating methylation biomarkers, demonstrate the potential for success and may encourage further investment in this domain.^[^ ^61^ ^]^ As of 2019, the World Health Organization (WHO) had granted prequalification to 89 IVD products, tailored for a range of diseases, in particular infectious ones. These include diagnostics for Human Immunodeficiency Virus (HIV), Hepatitis C Virus (HCV), Hepatitis B Virus (HBV), Malaria, Human Papilloma Virus (HPV), and G6PT enzyme deficiency. The range of these products covers Rapid Diagnostic Tests (RDT), Enzyme ImmunoAssays (EIA), and Nucleic Acid Tests (NATs). More specifically, 21 NATs, intended for diagnosing and monitoring diseases like HIV, HPV, and HCV, had been granted prequalification. This certification has facilitated the entry of numerous products into the global markets.^[^ ^62^ ^]^

**Figure 4:**
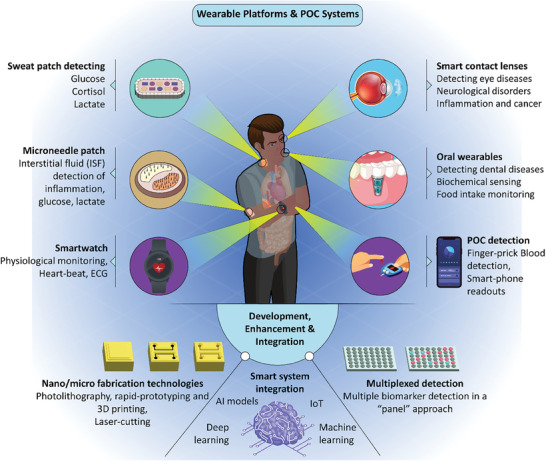
Illustration of wearable systems and POCT. Wearable technology platforms are currently being extensively investigated for their capability to detect biomarkers in various biofluids for POCT and on‐site operation by patients. This includes the use of wearable sweat collection/detection patches for sweat analysis, Reproduced with permission.^[^ ^85^ ^]^ Copyright 2021, Advanced Science published by Wiley‐VCH GmbH), dermal patches for Interstitial Fluid (ISF) analysis, Reproduced with permission.^[^ ^20b^ ^]^ Copyright 2022, Springer Nature Limited, oral wearables for saliva analysis, smart contact lenses for tear analysis, and smartwatch systems for physiological monitoring. The detection methodologies employed in these platforms encompass electrochemical, colorimetry, and optical transduction, with various readout modules such as potentiostat systems or optical light intensity detection, all techniques enabling miniaturized POCT. These point‐of‐care solutions are versatile in terms of their microfabrication technique, potential for integration with smart systems, as well as applicability for the detection of a panel of biomarkers through multiplexing.

The emergence of innovative mobile and wearable POCT systems is propelled by the growing demand for self‐testing options. Recent wearable POCT systems have been engineered to offer continuous health monitoring, integrating seamlessly into the daily activities of patients, most of which operate through real‐time and non‐invasive detection.^[^ ^71^ ^]^ These systems include a sample handling unit, a biosensor element, a signal processing unit, and software to communicate with off‐body devices. Configured as wearable bands or patches, such platforms offer non/minimally invasive sampling for on‐body biofluid analysis, transmitting data to off‐body devices for user monitoring or healthcare provider diagnostics.^[^ ^72^ ^]^ The integration of these sensors across various body locations, including the arm,^[^ ^73^ ^]^ forehead,^[^ ^74^ ^]^ chest,^[^ ^75^ ^]^ and back^[^ ^76^ ^]^ has been extensively researched to monitor biomarkers in sweat^[^ ^77^ ^]^ and Interstitial Fluid (ISF).^[^ ^78^ ^]^ Additionally, there has been significant progress in the development of ocular sensors like smart contact lenses for the detection of biomarkers in tears,^[^ ^79^ ^]^ aqueous humor,^[^ ^80^ ^]^ and vitreous samples,^[^ ^81^ ^]^ complemented by examples of oral wearables for saliva analysis.^[^ ^82^ ^]^ A critical factor in the development of such systems is the flexibility of the constituent materials and electronics, which is essential for enhanced wearability, comfort, and compatibility.^[^ ^83^ ^]^ This aspect is facilitated by recent advancements in printed electronics and material science.^[^ ^84^ ^]^ **Figure** **4** provides a comprehensive illustration of diverse configurations of wearable systems for physiological monitoring and biomarker detection.

**Figure 5:**
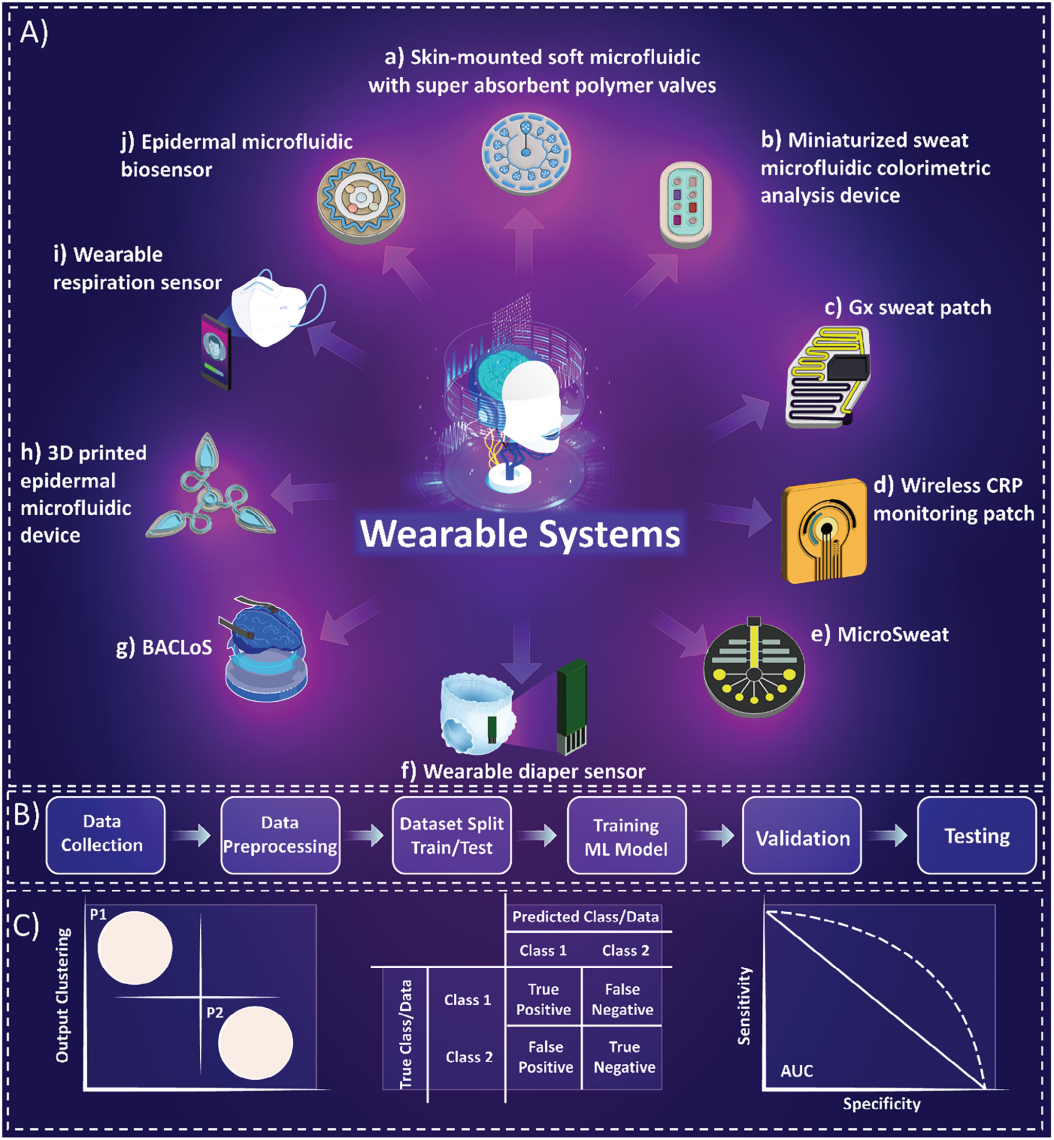
Wearable systems for biomarker detection and on‐site health monitoring. A, a) Colorimetric sweat chloride analysis enabled through skin mount microfluidic system featuring superabsorbent polymer valves, Reproduced with permission.^[^ ^114^ ^]^ Copyright 2018, John Wiley and Sons. b) Miniaturized microfluidics and colorimetric analysis for detection of nutrients in sweat and supplying vitamins, Reproduced with permission.^[^ ^85^ ^]^ Copyright 2021, Wiley‐VCH, c) The Gx sweat patch for personalized sweat rate determination and sweat chloride analysis for athletic use, Reproduced with permission.^[^ ^115^ ^]^ Copyright 2023, Springer Nature Limited. d) A sweat patch embedded with electrochemical sensors for C‐reactive protein (CRP) monitoring in sweat, Reproduced with permission.^[^ ^103^ ^]^ Copyright 2023, Springer Nature Limited, e) MicroSweat: A capillary microfluidic sweat collection patch for stress monitoring via determination of sweat cortisol levels, Reproduced with permissions.^[^ ^86^ ^]^ Copyright 2022, Wiley‐VCH. f) Electrochemical urine analysis via a wearable diaper sensor for urinary incontinence complications, Reproduced with permission.^[^ ^94^ ^]^ Copyright 2022, Elsevier. g) Wearable Electroencephalogram (EEG) device with a Brain–AI Closed‐Loop System (BACLoS) for predicting human cognitive consequences, Reproduced with permission.^[^ ^116^ ^]^ Copyright 2022, Springer Nature Limited. h) Microfluidics skin interface sweat analysis with a 3D complex structure enabling integration of colorimetric assays evaluating sweat chloride, Reproduced with permission.^[^ ^105^ ^]^ Copyright 2016, The American Association for the Advancement of Science. i) Respiration sensor in wearable format inside a mask for chronic kidney disease monitoring through ammonia (NH_3_) content measurement, Reproduced with permission.^[^ ^95^ ^]^ Copyright 2022, American Chemical Society. j) Soft wearable microfluidics for sweat capture, storage, and analysis using the smartphone‐assisted colorimetric technique, Reproduced with permission.^[^ ^106^ ^]^ Copyright 2016, The American Association for the Advancement of Science. B) The procedure of data analysis using AI algorithms from data collection, preprocessing, and dataset split to test and train, training the Machine Learning (ML) or Deep Learning (DL) model, validating the model, and final testing for predictions. C) The output of the wearable systems integrated with AI algorithms can be represented as clustering the data, determining the true or false negative/positive rates, and the accuracy of the model and analysis.

Wearable devices transform the paradigm of human‐computer interaction by introducing various forms and functions. These devices are predominantly utilized in the healthcare sector for monitoring vital physiological data. Moreover, they are instrumental in improving sports performance and fostering greater awareness of environmental factors.^[^ ^85^, ^86^ ^]^ Within this realm, wearable sensors play a pivotal role, particularly in medical contexts, where they aid in the monitoring of physical and chemical parameters, thereby elevating the standard of health care. Additionally, wearable sensors track body movements and environmental conditions, providing insightful information.^[^ ^87^ ^]^ A prime example of innovation in this field is the advent of smart contact lenses. These advanced wearable devices offer the capability for real‐time, non‐invasive detection of biomarkers, in addition to facilitating drug administration.^[^ ^88^ ^]^ They excel in measuring glucose levels in tear fluid, evaluating glaucoma via Intraocular Pressure (IOP) assessment, monitoring hypoxia and salt concentration levels, and administering specialized ophthalmic treatments.^[^ ^83^ ^]^ Their multifaceted, integrated configuration enhances data acquisition pertinent to the diagnosis of ocular diseases, spotlighting significant contributions to medical treatments. This includes accurate drug delivery and surgical procedure evaluation.^[^ ^89^ ^]^ In another example, sweat, as a biological secretion, carries biochemical information indicative of metabolic processes occurring within the body. This fluid contains electrolytes, hormones, proteins, nucleic acids, micronutrients, and other agents that reflect an individual's health status, stress levels, and dietary habits.^[^ ^90^ ^]^ The emergence of wearable sweat biosensors marks a significant stride in the domain of personalized health tracking. These devices analyze sweat at a molecular level, scrutinizing chemical biomarkers to offer real‐time assessments of physiological conditions. Innovations in sweat extraction methodologies, real‐time biosensing technologies, flexible material science, device integration, and wireless communication systems have culminated in the development of wearables that are not only lightweight, comfortable, and stylish but also economically viable and rich in informational value.^[^ ^91^ ^]^ An exemplary manifestation of this cutting‐edge technology is the development of wearable microneedles, which track physiologically relevant levels of glucose in the ISF and provide early warnings for potential diabetes development.^[^ ^92^ ^]^ These systems have also proven valuable in measuring lactate levels,^[^ ^20b^ ^]^ playing a crucial role in the diagnosis and management of critically ill patients. This is particularly vital in emergency scenarios, where they can be instrumental in forecasting the onset of septic shock. Additionally, they can be used to measure alcohol levels and offer a promising solution for continuous, quantitative alcohol content testing. These highly integrated sensors offer multiplexed functionality and are incredibly accurate when worn on the body, rendering them a valuable tool for everyday use by individuals.^[^ ^93^ ^]^ **Table** **2** provides a comprehensive insight into various wearable technologies devised in recent years for on‐site biomarker detection. **Figure** **5** depicts a schematic illustration of several wearable technologies for sweat, tear, breath, or urine analysis, along with pathways and methods for implementation of AI systems for data analysis.

**Table 2:** Overview of wearable systems for biomarker detection.

Platform	Sensing Technology	Biospecimens	Sensitivity‐LOD‐LOQ‐Linear range	Disease	Biomarker	Location on Body	Clinical Samples	References
Wearable Sensing System Based on Smartphone and Diaper	Amperometry	Urine	Sensitivity (Glucose): 22.1 nA/mm for a range of 0 to 2 mm Sensitivity (Glucose): 63.5 nA/mm for 2 to 20 mm Sensitivity (Uric Acid):1.92 µA/mm	ARF, Gout, and CVD	Glucose and UA	Diaper	1 Subject	[94]
Wearable Respiration Sensor	Resistive	Breath	LOD: 50 ppb	CKD	NH_3_	Face Mask	8 Subject	[95]
Uricase@MAF‐7‐Based EC Sensor	Amperometry	Sweat	LOD: 0.34 μm	Disease‐Related Metabolites	UA	Arm	10 Healthy Subjects	[96]
Vertical Fluidic‐Controlled Wearable Sensor Platform	Impedimetric	Sweat	Detection Range: 0.5–20 (µL)/(Min.Cm) For Sweat rate Detection Range: 1–200 mm For Total Electrolyte Concentration	Dehydration Status	Sweat Rate and Electrolyte	Forehead	7 Subjects	[97]
Wearable Microneedle‐Based EGT	EGT	ISF	LOD: 2.78 μm	DN	Sodium	Forearm	1 Subject	[98]
Wearable Hydrogel Patch	Amperometry	Sweat	LOD: 4 μm	Diabetes	Glucose	Palm, Back of Hand, and Finger	2 Healthy Subjects	[99]
Wearable Eye Patch Biosensor	Colorimetric	Tear	Semiquantitative pH: 5.4 – 8 Albumin: 0.5 – 10 g L^−1^ AA: 0.01‐12 mm glucose: 0.025 – 2.5 mm	Eye Health and Diabetes	pH Albumin AA Glucose	Under Eye	5 Healthy Subjects	[100]
Wearable Fluorescent Contact Lenses	Fluorescence	Tear	LOD: 9.3 μm	Diabetes	Glucose	Eye	On Rabbit	[101]
Smartphone Light‐Driven Zinc Porphyrinic MOF Nanosheets‐Based Enzyme‐Free Wearable Device	PEC	Sweat	LOD: 3.61 μm	Immune System Status	AA (Vitamin C)	Upper Arm	3 Healthy Subjects	[102]
Wearable and Wireless Patch	Potentiometric	Sweat	LOD: 8 pm	CD	CRP	Upper Arm and Forearm	3 Healthy Non‐Smokers 3 Healthy Smokers 3 Post‐Covid Subjects 1 Patient with COPD	[103]
Nanoporous, Heterogeneous, and Dual‐Signal Wearable	Both Colorimetric and Resistive	Breath	Detection Range in Colorimetric: 0−50 ppm Colorimetric with 4 different colors LOD (Resistive) = 0.14 ppm	CKD	NH_3_	Mouth and Nose Mask	None	[104]
Skin‐Interfaced Microfluidic Systems with Spatially Engineered 3D fluidics	Colorimetric	Sweat	–	Health Status	Chloride	Forearm	8 Healthy Subjects	[105]
Soft, Wearable microfluidic Device	Colorimetric	Sweat	Calibration Curves Lactate: 1.6 – 100 mm Glucose: 1.6 – 25 mm pH: 5–8.5 Creatinine: 15.6 – 1000 μm Chloride: 39 – 625 mm	Health Status	Lactate Glucose pH Creatinine Chloride	Lower Back and Volar Forearm	21 Subjects in 2 Tests	[106]
Skin‐Interfaced, Miniaturized Microfluidic	Colorimetric	Sweat	Calibration Curves: Vitamin C: 2 – 100 μm Calcium: 0.2 – 3 mm Zinc: 20 – 100 μm Iron: 20 – 100 μm	Nutrients Level	Vitamin C Calcium Zinc Iron	Lower Back, Stomach, and Upper Arms	7 Human Subjects	[85]
MicroSweat	ELISA	Sweat	Detection Range: 0.47– 200 ng ml^−1^	Stress	Cortisol	Lower Back, Upper Back, Forehead, Armpit, and Back of The Hand	3 Healthy Subjects	[86]
Wearable Breathing Sound Monitoring	ML (Fisher LDA)	Sound of Breathing	Sensitivity: 91.51% Positive Predictive Values: 100%	ORD	Wheezing Sound	Upper Right Anterior Chest Surface	952 Breathing Sounds	[107]
Wearable Sensor Data and Self‐Reported	DETECT Application Including ML Approach	ATD	AUC: 0.8 IQR: 0.73–0.86	COVID‐19	RHR, Sleep and Activity Metrics	Smartwatch + Self Reported Symptoms	30 529 Subjects	[108]
Wearable Sensor Devices Using Dynamic Warping Algorithm	ML (DTW)	Walking Pattern	Sensitivity: 95.9%, Specificity: 94%	AD	Foot Movement	Foot	223 Subjects	[109]
Wearable Sensor System Using an OECT	ECT	Sweat	LOD: 11 nm, Sensitivity: 1.9 mA/mm	Health monitoring	Lactate	Potentially on skin	None	[110]
Wearable Plasmonic‐Metasurface Sensor	SERS	Sweat	LOD: 10 nm	Drug Concentration	Nicotine	Forearm	6 Healthy Subjects	[111]
LEG‐CS	DPV	Sweat	LOD (Uric Acid): 0.74 μm, LOD (Tyrosine): 3.6 μm	Health Conditions	UA and Tyrosine	Forehead and neck	1 Subject	[112]
Wearable EC Glove‐Based Sensor	Voltammetry	Drug	LOD: 10 μm	OS	Fentanyl	Gloves (thumbs and index fingers)	None	[113]

AA: ascorbic acid, AD: Alzheimer's Disease, ARF: acute renal failure, ATD: activity tracker data, AUC: area under the curve, CD: chronic diseases, COPD: chronic obstructive pulmonary disease, CVD: cardiovascular disease, CKD: chronic kidney disease, CRP: c‐reactive protein, DN: dysnatremia, DPV: Differential pulse voltammetry, DTW: dynamic time warping algorithm, EC: electrochemical, ECT: electrochemical transistor, EGT: extended gate transistor, IQR: interquartile range, ISF: interstitial fluid LEG‐CS: laser‐engraved graphene‐based chemical sensor, LDA: linear discriminant analysis, ML: machine learning, NH_3__:_ ammonia, OECT: organic electrochemical transistor, ORD: obstructive respiratory disease, OS: opioids screening, PEC: photoelectrochemical, RHR: resting heart rate, UA: uric acid

When it comes to the regulatory practice for wearable systems and POC platforms, their clinical utilization on a large scale seems to be still in its early stages. However, during the COVID‐19 pandemic, the importance of having near‐patient tools that enable remote disease detection and monitoring has become even more significant, with many of the POC devices receiving Emergency Use Authorization (EUA), and later on clearance for clinical use. U.S. Food and Drug Administration (FDA), or other regulatory agencies, evaluate the medical devices in the POC and wearable format in terms of their user‐friendliness, safety, accuracy of results, as well as affordability.^[^ ^117^ ^]^ One example of a POC unit receiving EUA includes the Accula SARS‐CoV‐2 test^[^ ^118^ ^]^ which simplifies PCR testing using a cartridge card. Another important example beyond the infectious disease realm, is Abbott's i‐STAT TBI cartridge for rapid detection of concussion, with the test being done outside of the emergency department with lower complexity and high accuracy in 15 min.^[^ ^119^ ^]^ In the wearable physiological monitoring field, recently gadgets such as the Electrocardiogram (ECG/EKG) monitoring device by Quardio Core have been cleared by the FDA for use, all providing simple operation.^[^ ^120^ ^]^ All platforms designed and developed for on‐site POC detection of physiological conditions should also be compared to their centralized laboratory versions, in terms of complexity, and whether the assay has received Clinical Laboratory Improvement Amendments (CLIA) waived status or FDA clearance, for determining the pathway ahead for regulatory approval.^[^ ^121^ ^]^

### 2.2 Intelligent Technologies and Wearable POCs

The application of wearable biosensors has grown in popularity as they offer a non‐invasive approach to monitoring human physiological parameters through biological fluids like sweat, tears, and saliva. These sensors are engineered to be flexible and are integrated within multiple networks to continuously monitor biomarkers. The fusion of wearable platforms with AI systems, as illustrated in Figure 5, provides profound opportunities in diagnostic methodologies. **M**achine **L**earning (**ML**) algorithms are employed to interpret the data collected by these sensors to determine an individual's health status. The ML algorithms utilized must be interpretable by medical professionals and decision‐makers ensuring a comprehensive understanding of the decision‐making process. To achieve this, human knowledge and reasoning should be transparently integrated into the architecture of the DL system to regulate the learning and decision process. This approach can also reduce the sample size required to train the ML algorithms. The amalgamation of explainable machine learning with wearable electronics is vital in health monitoring and medical interventions.^[^ ^122^ ^]^ For instance, the use of ML in the healthcare industry, particularly in epilepsy management, is gaining momentum. One significant application of ML in this area is the detection and prediction of seizures using **W**earable **D**evices (**WDs**). However, not all currently available WDs employ ML algorithms. By implementing ML on data gathered from a substantial patient cohort using WDs, there is a potential to transform the diagnostic and therapeutic strategies in epilepsy care, for seizure detection and prediction via Electroencephalogram (EEG) DL analysis.^[^ ^123^ ^]^ To validate the efficacy of wearable biosensors and remote monitoring platforms that leverage ML, it is crucial to conduct randomized controlled trials with enough statistical power to substantiate their role in improving patient outcomes. Critical to this endeavour is the precise definition of interventions required for each clinical alert and the accuracy of ML‐driven alerts and prognostications. During the COVID‐19 pandemic, where efficacious treatment options are still limited, the implementation of clinical alerts can significantly aid in meticulous patient triage and in the judicious allocation of hospital resources.^[^ ^124^ ^]^

AI is a valuable tool for the identification, classification, characterization, and prediction of datasets derived from microfluidic systems. Integrating ML and AI into microfluidic devices harbours the significant potential for the creation of sophisticated monitoring systems in the future. This synergy facilitates an efficient setup and user‐friendly operation, thereby yielding significant time and resource efficiencies.^[^ ^125^ ^]^ AI also introduces new avenues for personalized treatment and observation of outcomes.^[^ ^126^ ^]^ Consider, for instance, a scenario wherein POC sensing technology, employed for COVID‐19 diagnosis, is seamlessly integrated with the Internet of Things (IoT) framework and empowered by AI techniques such as ML and DL, enabling the sophisticated analysis of the data and delivery of actionable insights. Such a synergistic approach enhances the efficacy of data storage, dissemination, and advanced analytical processing capabilities.^[^ ^127^ ^]^ Moreover, the confluence of ML, AI, biosensor technologies, and mobile communication devices empowers individuals to enhance self‐care, stay updated on their environment, and gain impactful insights regarding disease prevention and control.^[^ ^128^ ^]^ In parallel, the field of medical diagnostics is witnessing a significant transformation with the integration of image processing and ML into routine histopathology.^[^ ^129^ ^]^ As an exemplar, the research conducted by Feng et. al demonstrated an innovative application of AI‐enhanced colorimetric analysis. This innovative classifier exhibited exceptional accuracy in detecting glucose concentrations in urine samples, with an impressive ability to discern even slight variations in concentration.^[^ ^130^ ^]^

Smart technologies also play an important role in clinical data collection and analysis from wearable systems and POC platforms. Researchers are enhancing POC diagnostics by creating smart sensing components, including miniaturized transduction elements, interdigitated electrode‐based sensing chips, selective low‐level detection, and long‐lasting sustainability to meet patient care requirements.^[^ ^131^ ^]^ An illustrative example of the need for such advancements is observed in the field of oncology, particularly in early cancer diagnosis, where the current limitation lies in the inadequacy of comprehensive screening tools.^[^ ^132^ ^]^ There is a critical need for highly accurate POC devices capable of early and precise detection of cancer biomarkers at the patient's bedside. Recent advancements in nano‐ and microfabrication technologies, combined with various sensing platforms, have enabled rapid, cost‐effective, and reliable POC cancer diagnostics, offering reduced reagent use, shorter analysis times, portability, and enhanced multiplexed analysis for improved diagnoses and prognoses.^[^ ^133^ ^]^ During the COVID‐19 pandemic, one of the major challenges in managing the disease involved the accurate identification of the virus and the development of tests that are rapid, precise, and widely accessible. While Reverse Transcription Polymerase Chain Reaction (RT‐PCR) continues to be the benchmark for diagnosis, there has been a notable emergence of alternative molecular methodologies and immunoassays. These tests minimize the reliance on laboratory infrastructure and mass screening, thus streamlining the task of screening and detection.^[^ ^134^ ^]^ In the context of infectious diseases, particularly in resource‐constrained regions, the expeditious detection of such diseases is imperative for the provision of timely patient care and treatment. The formulation of a systematic approach for identifying specific biomarker profile characteristics of each disease is essential to improve future screening efforts. Owing to the symptomatic congruence observed across a spectrum of infectious diseases, there is a high demand for multifunctional, versatile POC diagnostic tests.^[^ ^135^ ^]^

Over the past decade, wearable, implantable, and continuous monitoring technologies have advanced, enabling the collection and analysis of longitudinal patterns rather than isolated biomarker values. This is especially beneficial for tracking chronic illnesses and overall well‐being.^[^ ^136^ ^]^ Owing to enhancements in biofluid sampling techniques, the integration of flexible materials, the advancement of wireless electronics, and the refinement of sensing platforms within wearable systems, great potential for commercialization and lab‐to‐market/clinic translation are evident. Their capability for the detection of multiple markers also enables a more accurate evaluation of the disease status and physiological conditions through multiplexing.^[^ ^137^ ^]^ Advancements in these areas have significantly improved their reliability, analyte monitoring capabilities, and wearability,^[^ ^78^ ^]^ with challenges yet to be addressed for enhanced performance. These challenges include the introduction and incorporation of novel, biocompatible materials, reliable and miniaturized power sources, with a high energy density and a long lifespan, and the invention of advanced wireless data transmission systems.^[^ ^138^ ^]^

## 3 AI Systems and Methods in Diagnostics

### 3.1 Role of AI in Medical Data, Image, and Signal Analysis for Disease Detection

**Figure 6:**
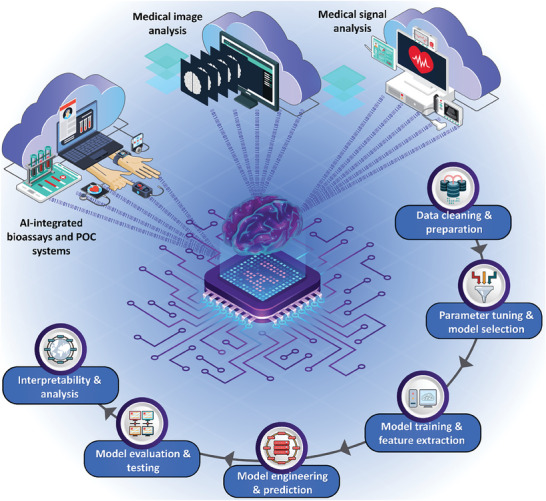
The role of AI in technologies for disease detection. AI systems are widely incorporated in the analysis of medical signals, as well as medical images, along with the design and analysis of bioassays and POC platforms. The methodology for analyzing systems using AI solutions, such as ML and DL methods involves data pre‐processing, labeling, selecting appropriate parameters, categorization of data, selecting the proper architecture, training the model, and testing and validation.

The rapid advancement of AI has revolutionized the field of diagnostics, offering great potential for improving accuracy, effectiveness, and accessibility. AI algorithms, including ML and DL, excel at processing vast amounts of biomedical data such as genomics, proteomics, medical imaging, and signals. By extracting relevant patterns and correlations from these datasets, AI accelerates the discovery of new biomarkers. This has been evident in many of the recent works on the utilization of smart technologies in clinical practice, such as DL‐based processing of medical images, e.g. Magnetic Resonance Imaging (MRI),^[^ ^139^ ^]^ Computed Tomography (CT)‐scans,^[^ ^140^ ^]^ or ultrasound images^[^ ^141^ ^]^ for discovering markers of various diseases such as neurodegenerative conditions.^[^ ^142^ ^]^ There is also an immense potential in integrating the POCT units, such as those developed for the detection of viruses during the pandemic, with AI‐driven data analytics, since these systems can be a boon for clinical diagnosis in developing nations, where centralized healthcare systems and resources are often limited.^[^ ^128^ ^]^ AI automation also facilitates the seamless integration of diagnostics into existing clinical workflows by enabling AI models to be integrated with Electronic Health Record (EHR) systems, or Electronic Medical Records (EMR),^[^ ^143^ ^]^ allowing for automatic data retrieval and analysis. This streamlined integration process eliminates manual data entry, reduces the risk of errors, and fosters interoperability between different healthcare systems, enabling efficient exchange of diagnostic information and improved care coordination.^[^ ^144^ ^]^ **Figure** **6** provides an overview of the applicability of AI solutions for the detection of disease biomarkers, as well as the procedure of data analysis and predictive AI model development.

**Figure 7:**
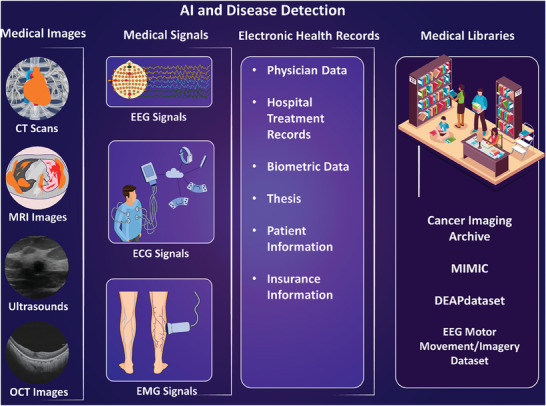
AI and disease detection through clinical and medical data, electronic health records, and medical libraries. The analysis involves examining medical images such as computed tomography (CT)‐ scans, for example for ML‐based examination of the Thoracic Aorta,^[^ ^155^ ^]^ Magnetic Resonance Imaging (MRI), for instance, to determine lung diseases,^[^ ^140b^ ^]^ ultrasounds,^[^ ^141^ ^]^ or Optical Coherence Tomography (OCT) imaging.^[^ ^156^ ^]^ Medical signals including EEG,^[^ ^157^ ^]^ Electrocardiogram (ECG),^[^ ^146^ ^]^ or Electromyography (EMG)^[^ ^147^ ^]^ can also be used as inputs of the AI algorithms. Evaluating the electronic medical health records such as physician data, hospital treatment records, biometric data, thesis, patient information, and insurance information, along with medical libraries including the Cancer Imaging Archive (TCIA),^[^ ^148^ ^]^ the Medical Information Mart for Intensive Care (MIMIC‐III) dataset,^[^ ^149^ ^]^ Physionet EEG Motor Movement/Imagery Dataset,^[^ ^143^ ^]^ and DEAP: A Database for Emotion Analysis using Physiological Signals,^[^ ^152^ ^]^ for AI‐based disease detection and prediction.

Analyzing medical data has been and is one of the constant challenges in the clinical domain, where artificial intelligence plays a significant role. Whether it be imaging modalities or medical signals such as EEG,^[^ ^145^ ^]^ ECG,^[^ ^146^ ^]^ or Electromyography (EMG),^[^ ^147^ ^]^ AI can be used for enhanced processing and data interpretation of results, automating the decision‐making process. Using a variety of existing medical libraries, AI models are trained to detect disease‐specific abnormalities and features, based on the expert‐labeled data as input. ML models often use medical libraries for this purpose. Having access to millions of pre‐registered and anonymized medical data creates robust reliable models that operate with high precision and accuracy. Some of these medical libraries which provide access to curated datasets for research and model development include, the Cancer Imaging Archive (TCIA),^[^ ^148^ ^]^ containing radiological images, pathology slides, and clinical data across various cancer types. Another significant resource is the Medical Information Mart for Intensive Care (MIMIC‐III) dataset, which includes de‐identified clinical data from Intensive Care Unit (ICU) patients, such as vital signs, laboratory results, and detailed clinical notes.^[^ ^149^ ^]^ For other types of medical signals, such as EEG, Physionet EEG Motor Movement/Imagery Dataset,^[^ ^150^ ^]^ CHB‐MIT Scalp EEG Database,^[^ ^151^ ^]^ the data collected from Children's Hospital Boston (CHB) and the Massachusetts Institute of Technology (MIT), or DEAP: A Database for Emotion Analysis using Physiological Signals^[^ ^152^ ^]^ can be utilized. The size of the dataset required for reliable AI methods varies depending on the complexity of the task. While larger datasets generally contribute to better performance, smaller specialized datasets with expert annotations can also yield reliable results. Various studies use different dataset sizes, for instance, 90 300 subjects for Alzheimer's disease,^[^ ^153^ ^]^ 129 450 images for skin cancer detection, or over 1700 X‐ray images for COVID‐19 detection from chest X‐rays.^[^ ^154^ ^]^ **Figure** **7** depicts the main types of clinical data used in the realm of AI analysis for the assessment of health conditions and disease detection, including medical libraries for predictive model development.

**Figure 8:**
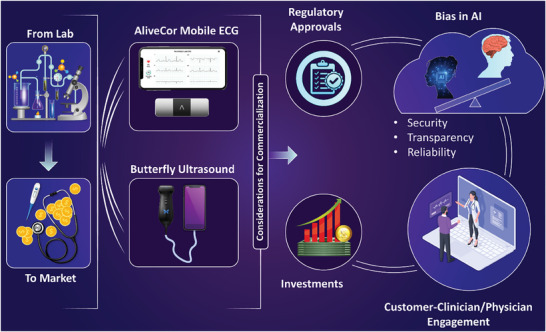
Commercialization of AI‐integrated diagnostic tools. Translation of the laboratory research products to the market, with examples including the AliveCor Mobile ECG system (Image reproduced with permission from AliveCor Inc.),^[^ ^161^ ^]^ and the Butterfly portable ultrasound system (Icon of Butterfly ultrasound probe provided with permission from Butterfly Network, Inc.).^[^ ^160^ ^]^ For this purpose, one should consider factors such as pathways for regulatory approvals, approaches for attracting investments, the possible bias in the AI algorithms that affect the security, transparency, and reliability of the data analysis, and interactions between the clinicians, patients, and other customers in the market.

Ethical and regulatory concerns grow increasingly relevant in the face of the commercialization of AI for analysis and diagnosis.^[^ ^158^ ^]^ While the use of AI in this field has been the topic of research for a long time, AI tools and products are being researched and commercialized more than ever because of recent developments in the field of AI and ML,^[^ ^159^ ^]^ such as stronger computing capabilities, data availability, creation of laws and regulations. A few examples of commercialized or near‐market AI‐integrated POC/wearables include the Butterfly Network's handheld, portable ultrasound device,^[^ ^160^ ^]^ with a DL neural network algorithm integrated for B‐line detection in lung ultrasound images. The AI algorithm will first evaluate the quality of the images before they undergo analysis. Another example is the AliveCor heart monitoring device,^[^ ^161^ ^]^ which performs electrocardiograms and analysis (**Figure** **8**). The product is used with a mobile application where users can see their data and assessments. AliveCor uses Deep Neural Networks (DNN) that take the recorded data as well as external factors (e.g., physical activity) to detect abnormalities in heart rhythm and assess cardiovascular health^[^ ^162^ ^]^ (Figure 8). There are many ethical and regulatory concerns with the use of AI for medical imaging and analysis. As AI systems in this domain are dynamic and continually evolving based on the data they receive, there is an increasing impetus for regulatory bodies to reconsider and update their strategies for regulating and approving AI‐enabled medical devices.^[^ ^163^ ^]^ For example, an AI algorithm can be biased if the training population is vastly different from the end‐user population; this can result in unfair and wrong diagnoses.^[^ ^163^ ^]^ Furthermore, there are concerns surrounding the security and availability of data used for training AI systems and conducting diagnoses. The strategies for storing and safeguarding patient data constitute critical elements in the regulatory discourse.^[^ ^164^ ^]^

Bias in AI systems used for medical imaging can manifest during both the data collection and model development phases. For example, if the training data disproportionately consists of images from a particular demographic group, the model may not effectively generalize to underrepresented groups. This imbalance can result in the model displaying varied diagnostic accuracies across different populations.^[^ ^165^ ^]^ An illustrative case is the variation in skin tones within dermatological image datasets, which can affect diagnostic accuracies for skin conditions among different racial groups. Lack of geographical representation in medical imaging data can also lead to varying performance across regions.^[^ ^166^ ^]^ In the phase of model development, factors such as flawed algorithm design, unrepresentative validation datasets, and improper threshold tuning can further entrench biases and disparities. For instance, setting an inappropriate threshold for detecting pneumonia in chest X‐rays could lead to different rates of misdiagnosis across groups.^[^ ^167^ ^]^ To address this, it is imperative to adhere to best practices in diversity and inclusion during the collection of training data. Employing techniques like data augmentation can enhance representation in datasets. Additionally, conducting rigorous testing on diverse validation sets is crucial for ensuring the reliability and fairness of the AI model. In the realm of medical devices, examples like pulse oximeters demonstrate reduced accuracy for patients with darker skin tones, a direct consequence of biases inherent in the training data. The acquisition of comprehensive clinical data across skin tones and extensive testing on historically underrepresented groups can help identify and resolve these disparities.^[^ ^166b^ ^]^ Gender imbalance in heart disease datasets can exacerbate biases within algorithms designated for ECG signal analysis, potentially resulting in diminished diagnostic accuracy for women. Balancing dataset gender ratios, along with specifically evaluating algorithmic performance in underrepresented subgroups is crucial for mitigating such biases.^[^ ^168^ ^]^ Improperly incorrect calibration of classification thresholds in AI models for chest X‐ray analysis may disproportionately affect underrepresented groups. Setting thresholds based on clinical needs and thoroughly examining model metrics across different subgroups are essential steps to prevent the introduction of discriminatory biases.^[^ ^169^ ^]^ **Table** **3** provides an overview of the latest AI systems and models for disease diagnosis.

**Table 3:** AI systems and models for disease detection.

Platform/System	Detection Modality	Disease	Type	AI Model	Data Size	Performance	References
Intelligent Model to Predict ELD	Online Available Data set – UCI ML Data Repository – Real Human Health Data	LD	Binary CL	ANN	65 532	Accuracy: 0.884	[170]
Multi‐Label Active Learning‐Based ML Model	Online Available Heart Disease Dataset – UCI ML Data Repository – Real Human Health Data	Heart Disease	Binary CL	Active learning Algorithms for multi‐label data	303	Accuracy: 57.4 ± 4% F1 Score: 62.2 ± 3.6%	[171]
ML for Emerging ID Feld Responses	One Million randomly sampled subjects (flu‐Like Illness) Extracted from the De‐identified NHI – Hospital Admissions Patients’ Health Dat	ID	Binary CL	Decision Tree, DNN, and LR	83 227	DNN is the best F1 Score of Sensitivity 85% Mean (95% Confidence Interval): 0.452 F1 Score of Sensitivity 90% Mean (95% Confidence Interval):0.444 F1 Score of Sensitivity 95% Mean (95% Confidence Interval): 0.422	[172]
SVM and x^2^ Statistical Optimal Feature Selection Model	Online Available Dataset – (UCI) Heart Disease Repository's Statlog and Cleveland Datasets – Real Human Health Data	Heart Disease	Binary CL	SVM	303 (Cleveland) 270 (Statlog)	Accuracy (Cleveland): 89.47% F1 Score (Statlog): 89.40%, ‐ Accuracy (Cleveland): 89.70% F1 Score (Statlog): 89.70%	[173]
Various ML CL technique	Online Available Dataset – ILPD Which Obtained from the UCI ML Repository – Real Human Health Data	LD	Binary CL	LR, GNB, DF, RF, Ada Boosting, GB, Extreme GB, Light GB, KNN, and Stacking	583	Light GB is the best Accuracy: 63% Precision: 63% Recall: 62% F1 Score: 63%	[174]
UL for Drug Discovery	Online Available Dataset – The ChEMBL and ZINC Natural Product Databases	AD	UL	NLP	19.9 million compounds	Identifying a Total of 18 Molecules	[175]
Diagnosing Long‐Term Risk of Older People by ML	Online Available Dataset – Kaggle – Real Human Health data	CVD	Binary CL	LR, NB, SVM, and RF	70 000	Logistic Regression is the best Accuracy: 72% Recall: 72% AUC: 72.1%	[176]
At‐Home Wearables and ML Sensitively Capture Disease Progression	Accelerometer Data from Wrist and Ankle‐Worn Wearable Sensors During Home Activities.	ALS	Binary CL	LinReg Models with L1‐Regularization	4637 sessions (24h) from 402 unique participants (376 ALS, 26 controls)	POT: 90%	[177]
Prediction Using ML‐Based on EMR	Data Collected of People with T2DM and 3 Years of Follow‐up at the PLA General Hospital. – Real Human Health Data^a)^	DKD	Binary CL	Light GB, Extreme GB, Ada Boosing, ANN, DT, SVM, and LR	816	Light GBM is the best AUC of 95% Confidence Interval (0.747–0.882): 0.815	[178]
SEG of the Thoracic Aorta with Congenital Valve Disease Using MRI	Cardiac MRI Datasets Acquired at CCHMC by 1.5 Tesla Clinical MRI Scanner	BAV	SEG	Total SEG	6	Dice Score (Heart Organ): 0.86 Dice Score (thoracic aorta): 0.72	[155]
Wearable Breathing Sound Monitoring	Clinical Study at Chang Gung Memorial Hospital – Breathing Sound	ORD	Binary CL	Fisher LDA	51 Clinical Study Including 952 Breathing Sou	Sensitivity: 91.51% PPV: 100%	[107]
Wearable Sensor Devices Implementing Dynamic Warping Algorithm	Gait Signals (Walking Patterns of Patients)	AD	Binary CL	DTW	223	Sensitivity: 95.9%, Specificity: 94%	[109]
Monitoring and Detecting AF Using Wearable Technology	Pulsatile PPG Captured Using a Multichannel Wrist‐Worn Device from Adult Patients who Were Hospitalized at the EUH, EUHM, and GMH.	AF	Binary CL	CNN + featuring beat‐to‐beat variability	98	AUC: 0.95 Accuracy: 91.8%	[179]
Wearable Fall Detector Using RNN	Online Available Dataset – SisFall Dataset – Waist Worn Accelerometer	Falling event	Multiclass CL (fall event or fall hazard or an activity of daily life)	RNN	117 000 Data From 38 Users	Sensitivity: 88.2%, Specificity: 96.4%	[180]
A Movement Decomposition and ML‐Based Fall Detection System Using Wrist Wearable Device	Experimental Study – Different Wrist Worn Sensors (Accelerometer, Gyroscope, and Magnetometer)	Falling event	Binary CL	KNN	792 Signals from 22 volunteers	Accuracy: 99% Sensitivity: 100% Specificity: 97.9%	[181]
N2Genetic‐nuSVM Algorithm	Online available Dataset – S‐ Alizade Sani Dataset – Contains Demographic, Symptom, Examination, ECG, Laboratory, and Echo	CAD	Binary CL	nuSVM	303	Accuracy: 93.08%, F1 Score: 91.51%	[182]
ML Based Predictors for COVID‐19 Disease Severity	Repository Contained Demographic, Clinical, and Laboratory data for All COVID‐19 Positive Patients Seen at the Keck Medical Center of USC, Verdugo Hills Hospital, and Los Angeles County, and USC Medical Center.	COVID‐19 disease severity	Twice Binary CL	RF	212	AUC (Predicting ICU Need): 0.80 AUC (Predicting the Mechanical Ventilation Need): 0.82	[183]
HRFLM	Online available Dataset – UCI Cleveland dataset – Real Human Health Data	CVD	Binary CL	HRFLM	297	Accuracy: 88.4%	[184]
Combined Model UL + SL	Endoscopic and Histological Data from Patients Recruited from the Genetics of Paediatric IBD Study at Southampton Children's Hospital.	PIBD	Binary CL	Coupling of PCA and MDS as UL + SVM as SL	284	Accuracy: 83.3%	[185]

AF: atrial fibrillation, ALS: amyotrophic lateral sclerosis, ANN: artificial neural network, BAV: bicuspid aortic valves, CAD: coronary artery disease, CCHMC: Cincinnati children's hospital medical center, CL: classification, CNN: Convolutional Neural Network, CVD: cardiovascular disease, DF: decision forest, DKD: diabetic kidney disease, DNN: deep neural network, DT: decision tree, DTW: dynamic time warping, ELD: early liver disease, ECG: electrocardiogram, EMR: electronic medical records, EUH: Emory university hospital, EUHM: Emory university hospital midtown, flu: influenza, GB: gradient boosting, GMH: Grady memorial hospital, GNB: gaussian naïve bayes, HRFLM: hybrid random forest with a linear model, IBD: inflammatory bowel disease, ID: infectious disease, ILPD: Indian liver patient dataset, KNN: k‐nearest neighbors, LD: liver disease, LDA: linear discriminant analysis, LinReg: linear regression, LR: logistic regression, MDS: multidimensional scaling, ML: machine learning, MRI: magnetic resonance Imaging, NB: naïve bayes, NHI: national health insurance, NLP: natural language processing, ORD: obstructive respiratory disease, PCA: principal component analysis, PIBD: paediatric inflammatory bowel disease, PLA: people's liberation army, POT: Power of detection, PPG: photoplethysmographic, PPV: positive predictive values, RF:random forest, RNN: recurrent neural networks, SEG: segmentation, SL: supervised learning, SVM: support vector machine, T2DM: type 2 diabetes mellitus, UCI: university of California, Irvine, UL: unsupervised learning, USC: university of southern California.

^a)^ Real human health data includes blood test data, physical exam data such as heart rate, and demographical data such as age, gender etc.

By identifying early disease indicators, AI systems can assist healthcare professionals in promptly detecting diseases, enabling timely intervention and tailored treatment plans. The applicability of such techniques is especially pronounced in the accurate diagnosis of time‐sensitive ailments, such as pneumonia induced by SARS‐CoV‐2, where delayed treatment can result in fatal outcomes. To this end, it has been established that the integration of biological assays, such as Reverse Transcription Loop‐Mediated Isothermal Amplification (RT‐LAMP), with AI systems can lead to improved outcomes.^[^ ^186^ ^]^ An additional key capability is characterizing early signs of the diseases through medical imaging. For instance, Jin et al.,^[^ ^187^ ^]^ deployed an AI system capable of automatically analyzing CT images to detect COVID‐19 pneumonia features. Besides, this system also automatically highlighted all lesion regions, facilitating a more rapid and efficient examination process. Furthermore, AI‐driven automation accelerates the diagnostic process by significantly reducing the time required for data analysis, interpretation, and reporting. This leads to faster diagnoses and timely medical interventions, particularly critical in point‐of‐care settings. For instance, the recent work of Owen et. al,^[^ ^188^ ^]^ describing Genome‐to‐Treatment is notable. This system is a virtual platform for automated diagnosis of genetic diseases and providing guidance on disease management. This approach employs expedited whole genome sequencing to achieve diagnoses within an average timeframe of 13.5 hours, showing enhanced performance in terms of analytics for structural and copy number variants. This platform is in particular of significant utility for children experiencing genetic diseases that progress at a high pace.

The application of AI in healthcare has achieved significant progress in areas such as medical imaging, disease diagnosis, and drug discovery. Yet, the potential of AI extends far beyond these areas. There is a substantial, largely unexplored opportunity for AI to make impactful contributions to the diagnosis and treatment of a wider array of conditions, including rare, infectious, and autoimmune diseases.^[^ ^189^ ^]^ In the case of infectious diseases, AI algorithms hold the potential to monitor and predict disease outbreaks, analyze genomic data of pathogens, and craft personalized treatment strategies. For instance, AI can assist in deciphering the underlying mechanisms of autoimmune disorders by analyzing vast amounts of molecular and genetic data, facilitating the discovery of novel therapeutic targets. To fully harness AI's potential in these domains, further research and development are needed to enhance AI algorithms, data quality, and regulatory frameworks, ensuring ethical and effective integration of AI technologies in healthcare.^[^ ^190^ ^]^

The FDA is actively engaged in differentiating AI‐enabled medical devices from traditional medical devices in terms of regulation oversight. The agency intends to adopt a new approach that is more adapted to evolving AI algorithms, allowing a more nuanced characterization and assessment of the associated risks. Central to this strategy is the requirement for increased transparency from manufacturers, coupled with the ongoing monitoring of devices and systems post‐approval.^[^ ^191^ ^]^ In the European Union, medical devices incorporating AI fall under the same regulations as other medical devices, pursuant to the Medical Devices Regulation (MDR).^[^ ^192^ ^]^ This regulation includes some provisions for software and algorithms intended for medical applications. However, the specific implications and applicability of these regulations to AI‐enabled medical solutions remain unclear. To create more explicit guidelines and regulations, the EU is actively formulating dedicated rules tailored to AI.^[^ ^193^ ^]^ A significant development in this context is the proposal of the Artificial Intelligence Act, which is presently progressing through the legislative process in the European Parliament. This act is comprehensive, encompassing all forms of AI technologies, inclusive of those used in the medical field.^[^ ^193b^ ^]^ Figure 8 also explores the influential factors in the realm of commercialization of research‐based AI diagnostics products, including the role of bias, regulatory approvals, investments, and interactions between patients and clinicians as end users.

### 3.2 AI and Biological Assays

Bioassays are the analytical methods to measure the concentration of a bioactive agent in relation to its efficacy in modulating cellular, tissue‐level, or biochemical processes.^[^ ^194^ ^]^ These assays entail the introduction of a stimulus — chemical, pharmacological, or physical —followed by the measurement of some response, quantified through a defined metric.^[^ ^195^ ^]^ Their application is pervasive in biological fields like pharmacology and genetics, signifying their status as a distinct scientific domain. In recent advancements, a variety of AI methods have been employed to increase the accuracy of measurements and to automate procedural tasks. Historically, a plethora of classical ML techniques have been extensively used,^[^ ^196^ ^]^ some of which are fundamentally grounded in statistical paradigms such as Bayesian inference and regression analysis.^[^ ^197^ ^]^ Today more modern methods like DL, Support Vector Machines (SVMs), and ensemble forest methods are being applied for the purpose of enhancing the data processing and outcomes of bioassays. ML models are capable of analyzing extensive datasets, discerning correlations even between seemingly unrelated information. This attribute is critically advantageous in the exploration and analysis of bioassays, where complex and multivariate interactions frequently occur. It also enables analyzing intricate datasets, especially those that possess high dimensionality. Various dimensionality reduction techniques like Principal Component Analysis (PCA) and advanced data engineering methods are used to find patterns and correlations, surpassing human capabilities in terms of speed and efficiency. This phenomenon is particularly evident in the context of gene expression experiments or other terms of high‐dimensional data.^[^ ^198^ ^]^ PCA specifically is utilized to create “principal” components, which are essentially linear combinations of the expressed genes and isolate the most significant element of the datasets with the highest variance. Empirical observations indicate that ≈95% of the variations can be found in less than a dozen principal components. Consequently, this allows for the exclusion of the remaining components, vastly lowering the dimensionality of the experimental data.^[^ ^199^ ^]^ Numerous recent studies have underscored the trend of integrating biological assays with AI algorithms, with **Table** **4** providing a concise overview of newly developed systems and platforms. In a pioneering study, an ML‐based label‐free SERS using 3D plasmonic Gold Nanoparticles (AuNPs) nanomembranes discriminates exosomes in human serum samples with 91.1% accuracy. The method, showcasing high sensitivity, diagnoses various cell lines, including cancer, without biomarker labeling. Dynamic SERS profiling monitors cancer cell chemotherapeutic processes noninvasively.^[^ ^200^ ^]^ Another study enhances Colorectal Cancer (CRC) diagnosis using exosomes. A 3D porous sponge microfluidic chip achieves 90% exosome capture efficiency. Deep mass spectrometry identifies CRC‐specific exosome protein, SORL1. A method using quantum dot labeling achieves exceptional diagnostic accuracy (AUC 0.99), outperforming conventional biomarkers. The system works well across early‐stage CRC, young CRC, and Carcinoembryonic Antigen (CEA)‐negative CRC patients.^[^ ^201^ ^]^ Addressing traditional Thyroid‐Stimulating Hormone (TSH) immunoassay challenges, a study introduces a single‐antibody biosensing platform. Raman spectral variations predict TSH concentrations with robustness, making it adaptable to diverse patient samples. Its simplicity and generalizability suggest potential widespread application in clinical settings.^[^ ^202^ ^]^ A novel Prostate Cancer (PCa) screening method utilizes urinary multimarker sensors and AI analysis. Prostate‐Specific Membrane Antigen (PSMA), Endoglin (ENG), Ets‐Related Gene (ERG), and Annexin A3 (ANXA3) biomarkers measured in urine using a Double‐Gate Field Effect Transistor (DGFET) biosensor achieve 100% accuracy in 23 individuals and 97.1% accuracy in blinded test panels with RadioFrequency Machine Learning (RFML). The multi‐marker sensing platform offers rapid and accurate PCa screening with the potential to reshape current paradigms.^[^ ^203^ ^]^

**Table 4:** State‐of‐the‐art AI‐enhanced bioassays for disease detection.

System Specifications	Assay Type	Target Markers/ Biomarkers	AI Model	Disease Studied	Clinical Samples	References
Urinary Multimarker Sensor	DGFET	Anxa3, PSMA, ERG, and ENG	RF and NN	Prostate cancer	76 Subjects	[203]
NA‐Based Activatable, AI‐Assisted Platform	Optical	SALL4	CNN	HCC	None	[204]
Diagnostic CNP‐Based Platform	Optical	BtT‐549, hcc 1806, and HCC 1143	ANN	TNBC	None	[205]
Universal Platform for Analyzing the Possible Existing State of two Protein Biomarkers	Optical	Avidin	–	Protein analysis	None	[206]
Photonic Crystal‐Enhanced Fuorescence Imaging Immunoassay Using machine learning	Fluorescent	Nt‐proBNP	PCA and SVM	Heart Failure	50 Subjects	[207]
3D Printed ECL imaging System Integrated with a Smartphone	ECL	Glucose and Lactate	LR, DT, RF, KNN, and AdaBoost	Diabetes	None	[208]
Multidimensional Digital Immunoassay Through MP‐Based Encoding and AI‐Based Decoding	CAT Immunoassay	CRP, PCT, and IL‐6	SVM	Inflammatory marker detection	30 Subjects	[209]
Ultra‐Rapid Nanoconfinement‐Enhanced Fluorescence Clinical Detection Platform Based on ML and DNA xerogel “probe”	Fluorescent	NGAL	ML	AKI	36 Subjects	[210]
AI‐Assisted Label‐Free SERS Biosensor (imeSERS)	SERS	5mC	ANN	Epigenetic DNA Methylations	None	[211]
AI‐Assisted Platform for Rapid Evaluation of Patient‐Specific Drug Sensitivity	Nds‐mediated MB‐based biosensor	SALL4	CNN	HCC	None	[204]
ML‐Assisted CB–GO/CP Electrode for Smart Portable Detection of Tyrosine	Electrochemical, Differential Pulse Voltametery	Tyr	ANN and SVM	Gastroesophageal Cancer	None	[212]
AI‐Based SERS of Exhaled Breath in Plasmonic‐MOF NPs	SERS	Gaseous Methanethiol	ANN	Oral Cancer	None	[213]
Methylscape Sensing Platform Using Cyst/AuNPs	Methylation‐Dependent DNA Solvation Using Cyst/AuNPs	5mC	RF and SVM	Leukemia	150 Subjects	[214]
Breathalyzer Platform for Fast Detection of SARS‐CoV‐2 Virion Particles	Electrochemical, Resistance	SARS‐CoV‐2	LSTM	SARS‐CoV‐2	15 Subjects	[215]
Exosome Enrichment Platform on a 3D Porous Sponge Microfluidic Chip	Fluorescent	CRC‐ Specific Exosome Membrane Protein, and SORL1	RF	CRC	None	[201]
Smartphone‐Based POCT Device for the Rapid Monitoring of Metabolic Biomarkers	Fluorescent	Cholesterol, Glucose, LA, and UA	ANN	–	16 Subjects	[216]
Single Antibody‐Based Biosensing Platform for TSH Test	SERS	TSH	ROBPCA, SMC	Thyroid	9 Subjects	[202]
SERS for Label‐Free Biofluid Interrogation	SERS	Cell Death Secretome	SVM	Cancer	None	[217]
SERS with 3D Plasmonic AuNPs Nanomembranes as Substrates	SERS	Exosome	SG, airPLS	Cancer	17 Subjects	[200]
Fluorescent Sensor Array Based on the Dual Coupling of a Nanoenzyme and Bioenzyme (horseradish peroxidase)	Fluorescent	Aβ40 and Aβ42	LDA	AD	None	[218]
Single Cell Sequencing and ML for Analysis of New Biomarkers in Sepsis	Single‐cell sequencing, GSEA, WGCNA	SNX3, NAIP, MMP8, EVL, TRBC1, BCL11B, FAIM3, ABLIM1, SIRPG, and CD7	RF, CIBERSORT, and Spearman	Sepsis	None	[219]
System for Predicting Glycaemia and Conttinious Glucose Monitoring with ML Algorithms and IoT tools	CGM	Glucose	SVM, RF	DM1	25 Subjects	[220]
PAD with a Smartphone Application Based on ML	Colorimetric	Glucose	LDA, GBC, RF	Diabetes	28 Subjects	[221]
Intelligent, Noninvasive Blood Glucose Monitoring System and Based on Smartphone PPG Signals	PPG	Glucose	MLC	Hyperglycemia	80 Subjects	[222]
Portable Planar Microwave Sensor Designed for Non‐Invasive Monitoring of Blood Glucose Levels	Portable Planar Microwave Sensor	Glucose	PCA	Diabetes	600 Subjects	[223]
IOMT Enabled Edge Device" named “Intelligent Glucose Meter” (iGLU)	PAS	Glucose	DNN	Diabetes	97 Subjects	[224]

5mC: c5‐methylcytosine, Aβ40: Amyloid Beta, Aβ42: Amyloid Beta 42, ABLIM1: actin binding LIM protein 1, AD: Alzheimer's disease, AdaBoost: adaptive Boosting, AI: artificial intelligence, air‐PLS: adaptive iterative reweighted penalized least‐squares algorithm, AKI: acute kidney injury, ANN: artificial neural network, ANXA3: annexin A3, BCL11B: b‐cell CLL/lymphoma 11B, BT‐549: breast cancer, CAT: computer vision‐based AI technology, CB–GO/CP: carbon black–graphene oxide conjugate polymer, CD7: cluster of differentiation 7, CGM: continuous glucose monitoring, CIBERSORT: Cell‐type Identification By Estimating Relative Subsets Of RNA Transcripts, CNN: convolutional neural network, CRC: colorectal cancer, CRP: c‐reactive protein, CNP: Carbon Nanoparticle, Cyst/AuNPs: cysteamine‐decorated gold nanoparticles, DGFET: dual‐gate field‐effect transistor, DB: diatom biosilica, DM1: type 1 diabetes mellitus, DNN: deep neural network, DT: decision tree, ECL: electrochemiluminescence ENG: endoglin, ERG: erythroblast transformation‐specific related gene protein, EVL: Enah/Vasp‐Like, FAIM3: fas apoptotic inhibitory molecule 3, GBC: gradient boosting classifier, GSEA: gene set enrichment analysis, HCC: hepatocellular carcinoma, IL‐6: interleukin‐6, IoT: internet of things, IOMT: internet of medical things, KNN: k‐nearest neighbour, LA: lactic acid, LDA: linear discriminant analysis, LinReg: linear regression, LSTM: long short‐term memory, ML: machine learning, MB: molecular beacon, MLC: machine learning classifier, MMP8: Matrix Metalloproteinase 8, MOF: metal organic framework, MP: microparticle, NA: nucleic acid, NAIP: neuronal apoptosis inhibitory protein, NB: nanobeacon, NDs: nanodiamonds, NGAL: neutrophil gelatinase‐associated lipocalin, NN: neural networks, NP: nanoparticle, NT‐proBNP: n‐terminal pro‐b‐type natriuretic peptide, PAD: paper analytical device, PAS: Photoacoustic Spectroscopy, PCA: principal component analysis, PCT: procalcitonin, POCT: point of care testing, PPG: photoplethysmography, PSMA: prostate‐specific membrane antigen, RF: random forest, ROBPCA: robust principal component analysis, SALL4: spalt‐like transcription factor 4, SARS‐CoV‐2: severe acute respiratory syndrome coronavirus 2, SERS: surface‐enhanced raman spectroscopy, SG: Savitzky–Golay algorithm, SIRPG: signal regulatory protein gamma, SMC: significant multivariate correlation, SNX3: sorting nexin3, SORL1: sortilin‐related receptor, Spearman: spearman's rank correlation, SVM: support vector machine, SVR: support vector regression, TNBC: triple‐negative breast cancer, TRBC1: t‐cell receptor beta constant 1, TSH: thyroid‐stimulating hormone, Tyr: tyrosine, UA: uric acid, WGCNA: weighted gene co‐expression network analysis.

Classical analytical approaches used in this field are mainly based on statistical algorithms such as expectation maximization, K means clustering, and k nearest neighbors, which were applied by Guan et al. to combine in vitro and in vivo bioassays to improve rat carcinogenicity prediction.^[^ ^225^ ^]^ Additionally, regression models like the Quantitative Structure–Activity Relationship (QSAR), which are predicated on the physical and chemical properties and molecular descriptions, are of paramount utility for the analysis of biological assays. Parallel to this, Support Vector Machines (SVM) have been extensively applied to analyze results derived from bioassay experiments, with applications extending to drug discovery and compound classification, as well as novel compound and property predictions.^[^ ^226^ ^]^ A multitude of unsupervised learning methods is of interest, primarily due to their ability to pick out patterns that are imperceptible to human observers and for their adeptness in accurately and swiftly categorizing samples lacking labels.

One interesting utility of smart technologies in the field of biological assays is the capabilities offered by computer vision to help track individual cells in order to automate research that necessitates cell tracking.^[^ ^227^ ^]^ Modern variations of this technology like the models developed by Harley et al. can now robustly track pixels even behind some occlusions and be trained in a matter of days.^[^ ^228^ ^]^ Presently, the application of computer vision has extended to various advanced methods of monitoring cells such as the end‐to‐end ML‐enhanced computer vision model developed by Soelistyo et. al,^[^ ^229^ ^]^ enabling visual interactions using convolutional and transformer attention models. Vall et al, as an example, employed transformers to predict novel bioassays using a chemical description encoding and a text description of the bioassay via the BioBERT model combined with Natural Language Processing (NLP) models to generate novel suggestions of new bioassays to predict viable new candidates for medications.^[^ ^230^ ^]^

**Figure 9:**
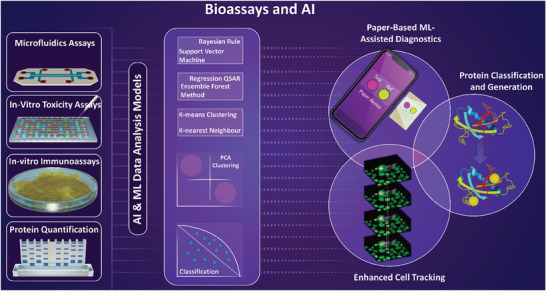
Bioassays and AI. Biological assays including microfluidic assays, in vitro toxicity assays, or immunoassays, as well as protein quantification such as western blotting can be integrated with statistical/ML methods for data analysis, including regression approaches such as Quantitative Structure‐Activity Relationship (QSAR), k‐means clustering, and k‐nearest neighbours, ensemble forest methods, Support Vector Machine (SVM), and Principal Component Analysis (PCA) with applications in enhanced cell tracking, Reproduced with permission.^[^ ^229^ ^]^ Copyright 2023, Frontiers, image processing for paper‐based microfluidics, Reproduced with permission.^[^ ^232^ ^]^ Copyright 2021, American Chemical Society, and protein classification/generation.

ML algorithms excel at analyzing complex images generated by bioassays, such as microscopy,^[^ ^231^ ^]^ histology, colorimetry, or radiology images. Khanal et al., for instance, used a DNN and compared it to other classical methods to accurately predict analyte concentrations via paper‐based microfluidics. This technique enabled the conversion of cell phones and similar devices into high‐accuracy colorimeters, producing results with notable precision.^[^ ^232^ ^]^ In biomedical image processing, these models demonstrate the capability to detect features, segment regions of interest,^[^ ^156^ ^]^ classify various cell types,^[^ ^233^ ^]^ and aid in the identification of anomalies,^[^ ^234^ ^]^ while supporting automated and high‐throughput image analysis. Generative models are also a new but exciting area of ML, where models can generate new original ideas albeit based on their trained dataset. To this end, graph networks are being used for protein folding^[^ ^235^ ^]^ and synthesis research,^[^ ^236^ ^]^ showcasing their versatility for any data that can be conceptualized and reduced to a graph format. **Figure** **9** illustrates different formats of bioassays that demand statistical or ML data analysis for the interpretation of results or development of predictive models, with examples in image processing for paper‐based diagnostics, cell tracking, or protein classification and generation.

**Figure 10:**
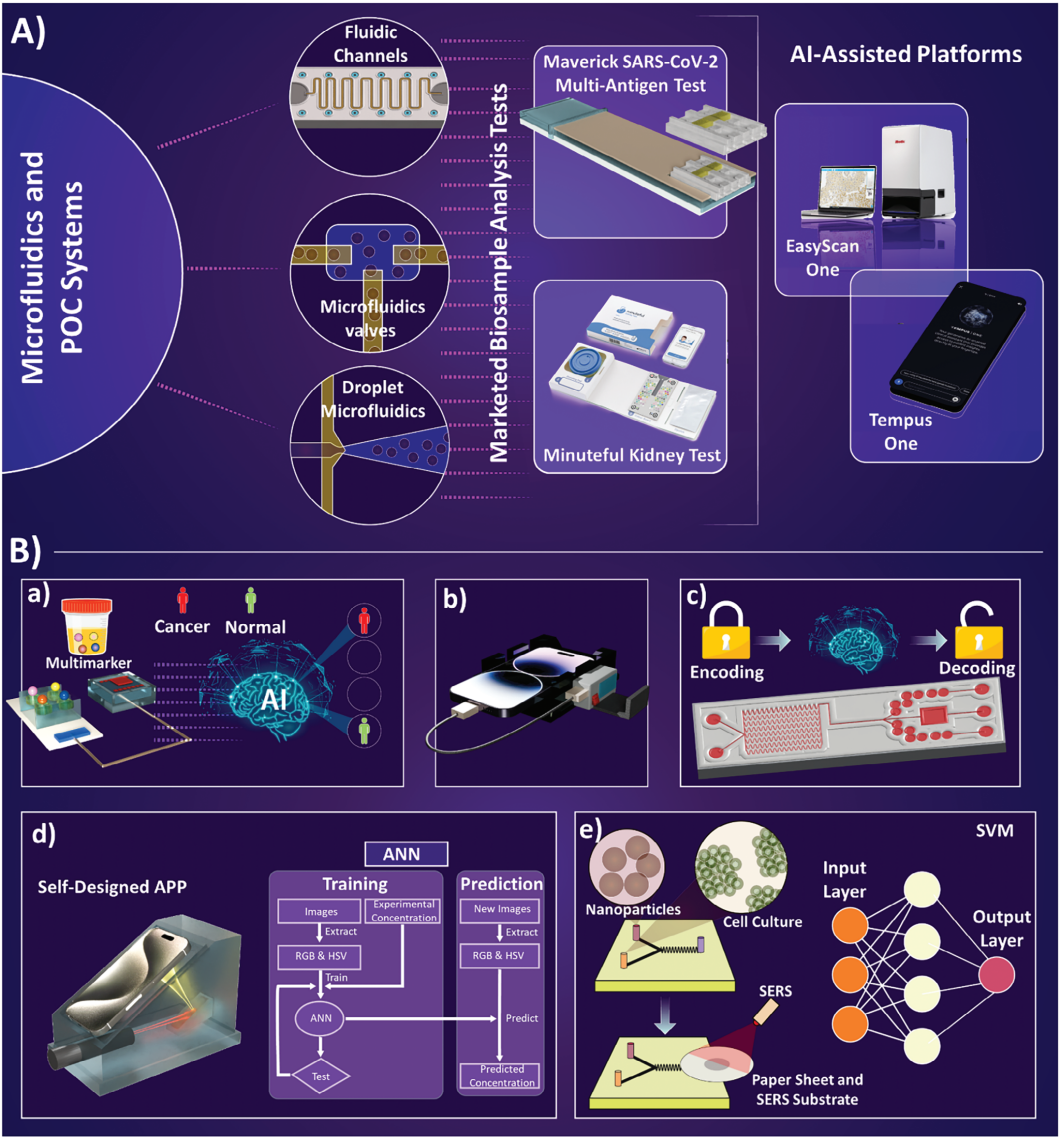
Integration of microfluidics in the POC systems and platforms for conducting biological assay with AI technologies. A) Various microfluidics elements such as microchannels, valves, and droplet microfluidic systems that can be used for developing platforms to conduct biological assays. Such techniques coupled with sensing modalities enable on‐site biosample analysis with marketed and FDA‐authorized examples including the Maverick™ SARS‐CoV‐2 Multi‐Antigen Serology Panel ^[^ ^244^ ^]^(Image reproduced with permission from Genalyte Inc.), or the Minuteful smartphone‐powered kidney test (Image reproduced with permission from Healthy.io Ltd).^[^ ^245^ ^]^ The role of AI systems in biological assays and clinical decision‐making is also becoming more established with marketed technologies such as EasyScan One (the newer version of EasyScan Go), a microscopy system for Malaria detection based on machine learning ^[^ ^246^ ^]^(Image reproduced with permission from Motic Instruments Inc), and the Tempus one system, one of the latest innovations of the Tempus labs incorporating generative AI solutions in precision medicine (Image reproduced with permission from Tempus).^[^ ^243^ ^]^ Recent research works explore novel applications of the integration of AI systems into the biological assays and biomarker detection systems, including a) AI‐assisted urinary multimarker sensor for prostate cancer screening, Reproduced with permission.^[^ ^203^ ^]^ Copyright 2021, American Chemical Society. b) Electrochemiluminescence biosensor with smart‐phone integrated and machine‐learning assisted algorithm for detection of various metabolites, Reproduced with permission.^[^ ^208^ ^]^ Copyright 2023, Elsevier. c) A microfluidic digital immunoassay for inflammatory markers and antibiotics detection empowered by a computer vision‐based AI‐mediated encoding‐decoding system, Reproduced with permission.^[^ ^209^ ^]^ Copyright 2023, American Chemical Society. d) Utility of the artificial neural network in processing the light parameters of the fluorescence for optical POC solutions, Reproduced with permission.^[^ ^216^ ^]^ Copyright 2023, Elsevier. e) High‐throughput SERS‐based classification of the cell secretomes assisted by the machine learning algorithms, Reproduced with permission.^[^ ^217^ ^]^ Copyright 2023, Wiley VCH.

Of interest in bioassays, there is currently research emphasis on the automation of circuit designs for potential application in on‐chip experiments,^[^ ^237^ ^]^ including microfluidic systems (**Figure** **10**). Moreover, the application of generative models extends to the strategic planning and structuring of experimental frameworks as a whole. Whether in part or fully integrated sections of experiments, Large Language Models (LLM), can be used to generate not only research directions but detailed designs of full experimental protocols. Recently, a model called algorithm for Physical Experiments based on nonparametric Ranking and Clustering (ALPERC) was combined with a random forest algorithm to conduct a case study on amorphous metallic alloys.^[^ ^238^ ^]^ Previous examples of this utility include the work of Clark et al., wherein ML techniques were employed to accurately annotate semantic descriptions of bioassays.^[^ ^239^ ^]^ The utilities of generative models, not only in the realm of bioassays but overall, for scientific inquiry and investigation are now being meticulously scrutinized.^[^ ^240^ ^]^

The integration of AI into bioassays holds the potential to significantly revolutionize the landscape of POC devices. Several enterprises are looking at developing POC systems enabled with AI to offer users a faster, smoother, and simpler testing process. A case in point is “Genalyte” (Figure 10), which specializes in the creation of POC solutions for bioassays and diagnostics. While their portfolio is not exclusively focused on AI‐enabled technologies, they have incorporated ML and AI algorithms into some of their solutions, and their platform has been evaluated for SARS‐CoV‐2 multiantigen serology assay.^[^ ^241^ ^]^ As another example, EasyScan Go (Figure 10), an AI‐enabled malaria detection platform developed by Motic, utilizes a microscopy system capturing images of magnified blood smears, subsequently leveraging ML algorithms to analyze the data, thereby providing an accurate diagnosis.^[^ ^242^ ^]^ AI systems are also finding applications as clinical assistants; Tempus, a biotechnology company developing AI‐integrated medical diagnostic solutions, is gaining recognition in the field of oncology. Oncologists can use the Tempus ONE, a device equipped with a proficient AI assistant. The portable device analyzes patient data, contextualizes it within the patient's health record, and utilizes external data from other, similar patients to compare and find possible similarities or trends. The device can also run checks and analytical tests upon the request of a physician.^[^ ^243^ ^]^ Currently, there are few AI‐enabled POC systems on the market. However, the integration of AI for bioassays and diagnosis has an important effect on the medical world. AI can perform some testing and diagnosis independent of the presence of medical professionals. As healthcare systems are becoming increasingly saturated, the use of AI can help alleviate the strain on hospitals and clinics. This would also allow medically underserved communities with limited resources, e.g., rural communities, to have access to testing that they would not have otherwise. Given the potential, many biotechnology companies are now increasingly researching AI‐assisted POC systems.

## 4 Progresses in Biomarker Discovery Using Novel Tools and Methods: From Wearable POC Implementation to Adoption of Intelligent Systems

### 4.1 Methods and Platforms for Biomarker Discovery Based on Medical Data

**Figure 11:**
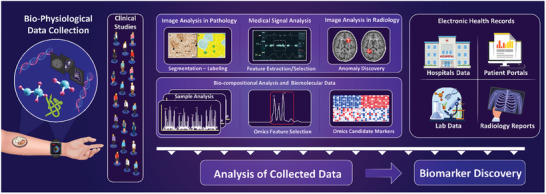
Biomarker discovery via wearable systems, microscopy and medical image analysis, signal analysis, omics, and Electronic Health Records (EHR). Clinical studies are conducted for collecting bio‐physiological data, either in a wearable format (Reproduced with permission.^[^ ^20b^ ^]^ Copyright 2022, Springer Nature Limited) or via imaging, microscopy, or medical signal acquisition. The acquired data include pathological microscopy images requiring segmentation/labeling (Reproduced with permission.^[^ ^250^ ^]^ Copyright 2012, American Association of Neuropathologists, Inc.) medical signals needing feature extraction as specific disease markers, radiology images for anomaly discovery, or biocompositional data analysis such as the outputs of the omics assessments including Liquid Chromatography‐Mass Spectroscopy (LC‐MS) or proteomics heatmaps. The EHRs such as data from hospitals, patients, lab tests, and radiology reports can also be used for introducing specific disease biomarkers in this realm upon analysis of collected data.

The recognition of biomarkers for disease detection and diagnosis is presently limited to a finite set. However, there is a growing emergence of new biomarker discovery initiatives aimed at detecting markers that may be further characterized and validated for their translational utility.^[^ ^247^ ^]^ Wearable chemical sensors provide a compelling avenue for addressing the specificity challenge inherent in biomarkers, allowing for concurrent monitoring of a diverse range of molecular signatures and the identification of temporal patterns. This approach offers an efficient and cost‐effective alternative to the conventional trial‐and‐error method of testing biomarker concentrations across various diseases.^[^ ^21^, ^248^ ^]^ A wearable chemical sensor for continuous real‐time analysis of a wide range of molecular biomarkers, including DNA, RNA, hormones, proteins, and viruses, holds significant promise for advancing public health monitoring and surveillance through reagent‐free, on‐body detection^[^ ^21a^ ^]^ (**Figure** **11**). Wearable devices can employ electrochemical sensors, which involve the immobilization of bioreceptor molecules (e.g., enzymes, antibodies, DNA, aptamers) on conductive electrodes for selective interaction with target molecules, providing real‐time, highly sensitive conversion of analyte concentration into electrical signals, or optical sensor systems, which modify optical detection elements through chemical or biological reactions, altering light absorbance or emission. These systems offer skin integration without extra components, yet achieving high sensitivity, stability, and frequent data collection in miniaturized wearables remains challenging.^[^ ^21^, ^101^ ^]^

EHRs can also serve as an important resource for biomarker discovery, particularly within complex patient conditions, offering a wealth of longitudinal data that expands research cohorts beyond the constraints of small sample sizes (Figure 11). This longitudinal patient data also enables the identification and validation of biomarkers over a “pre‐diagnosis” timeframe, facilitating the early detection of disease indicators and informing preventive interventions. EHRs, when coupled with AI, play a pivotal role in disease diagnostics, such as heart failure^[^ ^249^ ^]^ and Alzheimer's,^[^ ^250^ ^]^ through the application of AI‐driven pattern detection and image recognition techniques. The integration of diverse health variables within EHRs, alongside the transformation of structured and unstructured data, creates a comprehensive clinical context that is instrumental in the development and implementation of ML algorithms and statistical models, ultimately advancing biomarker discovery.^[^ ^251^ ^]^

The identification of new biomarkers can also be achieved through the utilization of imaging biomarkers, which involves the visual inspection of images or the spatial profiling of biomolecules directly from tissue sections. This approach aids in the discovery of imaging biomarkers that hold potential for diagnostic purposes and the identification of candidates for disease biomarkers.^[^ ^252^ ^]^ For instance, the identification of distinctive patterns within pathology images from two Antibody‐Drug Conjugates (ADC) lung cancer patients—one with a poor survival outcome and another with a longer lifespan—can serve as a blueprint for future ADC lung cancer detection strategies.^[^ ^253^ ^]^ Nevertheless, the manual search for such imaging biomarkers is labor‐intensive and impractical for analyzing a large number of samples. Consequently, the development of automated image analysis tools is imperative to assist pathologists in the identification of disease‐specific imaging biomarkers (Figure 11).

**Figure 12:**
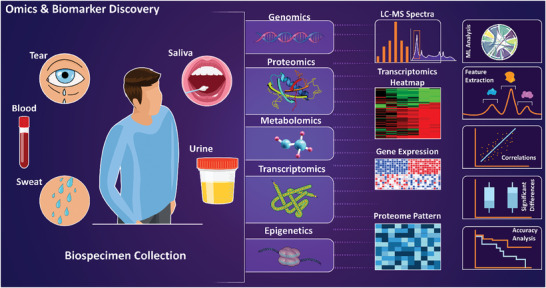
Omics and Biomarker Discovery. Biocompositional analysis of various biosamples including blood, saliva, tear, urine, and sweat through genomics, proteomics, metabolomics, transcriptomics, and epigenetics leads to the introduction of novel disease markers upon analyzing the generated chromatography spectra, transcriptomics heatmaps, gene expressions, and proteome patterns. This includes AI, ML, or DL analysis, extracting features associated with disease‐specific biomolecules, extracting correlations and significant differences in intensities/concentrations, and analysis of accuracy.

ML employs sophisticated algorithms to autonomously identify patterns in diverse datasets, making it particularly effective in biomarker research across various diseases, from proteomics to imaging data. It utilizes mathematical methods to train models for specific tasks, with classification and feature selection being key techniques in biomarker discovery (**Figure** **12**).^[^ ^247^ ^]^ Feature selection aids biomarker studies by enhancing interpretability and potentially improving classification accuracy, particularly in dermatology, where it has been applied to develop predictive models for various skin diseases based on different biomarkers. This method, for instance, falls into three categories: filter, wrapper, and embedded methods, all ultimately identifying informative features as potential biomarkers.^[^ ^254^ ^]^ Another ML technique, classification, is also valuable in biomarker discovery as it assesses potential biomarkers identified by other methods, utilizing the distinct characteristics of treated and control groups to classify samples and determine their informativeness in distinguishing groups. This supervised learning approach involves labeled samples represented by feature sets.^[^ ^254a^ ^]^

**Table** **5** provides a concise overview of recently developed systems at the intersection of AI and omics, highlighting pioneering technologies that have significantly advanced biomarker discovery. For example, Sonja et al. introduced a novel approach called Deep Visual Proteomics (DVP). Automated microdissection is combined with AI systems based on image analysis in this study, in tandem with sensitive mass spectrometry. The Colorectal Adenomas (CRA) tissues were first fixed with formalin and embedded with paraffin before staining with Caudal‐type homeobox 2 (CDX2), which is a specific colorectal cancer protein. The integration of the DVP with the procedure of pathology process, assisted in detecting several markers of disease reccurance including Deleted in Malignant Brain Tumors 1 (DMBT1), Myristoylated Alanine Rich C‐Kinase Substrate (MARCKS), and Cluster of Differentiation 99 (CD99). The outcomes of the study emphasize the capabilities of spatial proteomics in refining early detection and contributing to precision medicine strategies, offering new possibilities for metabolic reprogramming in CRA.^[^ ^255^ ^]^ In another study, an accurate prediction model was proposed using clinical and biomarker data. In a population of 889 patients under coronary angiography practice, 109 biomarkers were measured in blood samples using Luminex xMAP technology. Procedural Acute Kidney Injury (AKI) was defined within 7 days after contrast exposure. Using ML techniques for identifying the predictors, and through employing the Least Absolute Shrinkage and Selection Operator (LASSO) a prognostic model was created. 4.8% of the patients experienced procedural AKI, and the final model included six predictors. Factors such as a previous diabetes diagnosis, the ratio of blood urea nitrogen to creatinine, concentration of C‐reactive protein, and level of osteopontin have shown to be positively correlated with AKI risk. The level of CD5 antigen‐like and Factor VII were demonstrated to be negatively correlated with AKI risk. A cross‐validated Area under the Receiver Operating Characteristic (ROC) curve (AUC) of 0.79 and an in‐sample AUC of 0.82 were used to illustrate the high accuracy of the model, which indicates a robust ability to predict procedural AKI. The findings highlight the potential of a proteomics‐supported biomarker model with clinical utility for accurate prediction of procedural AKI occurrence in patients going through coronary angiography.^[^ ^256^ ^]^ Huang et al. established an AI model based on fecal multi‐omics data. The examination involves a clinical group of 299 individuals, incorporating 86 individuals in good health, 140 individuals diagnosed with Crohn's Disease (CD), and 73 individuals diagnosed with Ulcerative Colitis (UC). Employing such omics data, ranging from metagenomics to viromics, proteomics, and metabolomics, the study utilizes feature engineering techniques to evaluate the importance, collinearity, and other relevant aspects of the features. The results reveal the establishment of three distinct diagnosis models that are simplistic in terms of features while presenting a high accuracy. The optimized feature set creates three classes of diagnosis model, representing an AUC of 0.83, for in tandem classification of healthy condition, CD, and UC. Additionally, hierarchical modeling for different self‐evaluation groups results in two models for populations with distinct self‐evaluations. Another three‐classification diagnostic model (AUC = 0.85) was also developed for the “very well” population. On the other hand, the “slightly below par” population model includes 22 features, with a slightly lower AUC of 0.84. It is worth noting that metabolomics and meta‐transcriptomics were the only features included in the optimized feature sets. This study provides a non‐invasive method for the diagnosis of Inflammatory Bowel Disease (IBD) patients and the detection of its subtypes, offering a simpler alternative to clinical colonoscopy and biopsy procedures through fecal sampling.^[^ ^257^ ^]^

**Table 5:** Recently developed AI‐driven omics biomarker discovery.

System specifications	Type of Omics Technology	AI model	Target Disease	Number of Samples	Assay Type	References
Use of ANNs in Predicting Ploidy Based on a Combination of Embryo Protein Profiles and Automatic Scores Assigned by Time‐Lapse Videos	Proteomics	ANN	Ploidy	81	PEA	[263]
DVP proteomics	Proteomics	DVP	CRA	9	Spectrometry	[255]
Non‐Targeted/Targeted Metabolomic and Semi‐Quantitative Immune‐Based Proteomic	Metabolomics, Proteomics	AI, ML	EC	440	–	[264]
AI‐Driven Approach for High‐Quality Full‐Chain Structure Predictions on the Pan‐Proteome of the Type Strain Acidithiobacillia	Proteomics	DeepFRI	Acidithiobacillia Mediated Biohydrometallurgy	129	–	[265]
Luminex xMAP Technology to Measure 109 Biomarkers in Blood from Patients Undergoing Coronary Angiographic Procedures	Proteomics	LARS and LASSO	AKI	889	Luminex xMAP Technology	[256]
AI Model Based on Fecal Multi‐Omics Data for the Multi‐Classification Diagnosis	Multi‐Omics Data (Metagenomics, Metatranscriptomics, Proteomics, Metabolomics, and Viromics, Faecal Calprotectin)	SVM	IBD	299	–	[257]
XGBoost Algorithm to Establish a Novel Diagnosis Model	Urinary Proteomics	XGBoost	CKD (IgA Nephropathy, Membranous nephropathy, DKD)	134	Spectrometry	[266]
Comparison of Data from PET/CT with Results from Panscopy with Biopsy and US‐FNAC for Patients Suspected of Head and Neck Malignancy	Genomic, Transcriptomic, and Proteomic	AdaBoost, ANNs, DT, XGBoost,and SVM	SCCHN	42	–	[267]
Use of AlphaFold, and Advances in LLMs	Proteomic	Bepler, esm1b, and RF	Lymphoma‐Derived Ig	45	–	[268]
Network‐Based AI Framework for Integrating Multi‐Omics Data, GWAS Findings, Protein‐Protein Interactome Networks, and Drug‐Target Networks	Multi‐Omics	Network‐Based AI	AD	–	BCA Protein Assay Kit	[269]
“HART PAD panel” Which Consists of One Clinical Variable and Concentrations of Six Biomarkers	Proteomics	Monte Carlo	PAD	354	Midkine, Kidney Injury molecule‐1, IL‐23, Follicle‐Stimulating Hormone, Ang‐1, and Eotaxin‐1	[270]

AD: Alzheimer's disease, AdaBoost: adaptive boosting, AI: artificial intelligence, Ang‐1: Angiopoietin‐1, ANN: artificial neural network, AKI: acute kidney injury, BCA: bicinchoninic acid CKD: chronic kidney disease, CRA: colorectal adenomas, DKD: diabetic kidney disease, DT: decision tree, DeepFRI: Deep functional residue identification. DVP: deep visual proteomics, EC: endometrial carcinoma, GWAS: genome‐wide association study, IBD: inflammatory bowel disease, IL‐23: interleukin‐23, LARS: least‐angle regression, LASSO: least absolute shrinkage and selection operator, LLM: large language model, ML: machine learning, PAD: peripheral artery disease, PEA: proximity extension assay, PET/CT: positron emission tomography/computed tomography, XGBoost: extreme gradient boosting, SCCHN: squamous cell carcinoma of the head and neck, SVM: support vector machine, xMAP: x multiplexed analyte profiling

The emerging discipline of omics‐centric methodologies, encompassing genomics, transcriptomics, proteomics, and metabolomics, presents a promising pathway for the biomarker discovery, diagnosis of diseases, and investigation of latent determinants that impact disease progression (Figure 12). The integration of proteomics studies with other omics domains yields a wealth of molecular information amenable to computational analysis, thereby augmenting the discernment of biomarkers. In recent years, global translational research has embraced multi‐omics methods utilizing patient samples, which involve a comprehensive analysis of combined data from diverse omics techniques. This integrated approach, yielding extensive datasets compared to single analyses, offers valuable insights into the complex origins of diseases, thus playing a pivotal role in disease diagnosis and treatment development. Consequently, the ongoing utilization of multi‐omics approaches, amalgamating various omics datasets, is poised to exert a substantial influence on translational research, particularly in fields like cancer biology, and will likely serve as the foundation for studying various diseases in the future. These innovations, conjoined with AI, have revolutionized disease‐oriented research by facilitating high‐throughput inquiries across diverse biological realms, thereby deepening our comprehension of maladies such as cancer, cardiovascular disorders, heart failure, and Alzheimer's disease.^[^ ^25^, ^258^ ^]^ For instance, DL is gaining attention in Alzheimer's disease research for analyzing unstructured data, particularly in image‐related data like MRI and Positron Emission Tomography (PET) scans. DNN and Deep Belief Network (DBN) are among the DL methods used to study AD expression activities and proteomics data, outperforming traditional ML methods in some cases.^[^ ^258a^ ^]^

### 4.2 Omics and Biomarker Discovery

Omics‐based strategies for biomarker discovery encompass four primary modalities. First, microRNA transcriptomics entails the examination of short RNA molecules governing gene expression, demonstrating promise in the identification of disease biomarkers by scrutinizing miRNA expression across various pathological conditions (Figure 12).^[^ ^259^ ^]^ Second, proteomics, a comprehensive study of the entire protein complement encoded by the genome, is conducted. This technique mainly harnesses mass spectrometry in conjunction with advanced bioinformatics, allowing for the identification and quantification of proteins linked to diseases. Significantly, proteomics has held promise for translation into clinical applications.^[^ ^260^ ^]^ Thirdly, neuroproteomics (specifically for brain injuries) is dedicated to the exploration of the protein complements within the genome, primarily within the central nervous system. Impressively, neuroproteomics has exhibited its utility in animal models and holds the potential for translation into clinical settings. Finally, metabolomics, which delves into the assessment of metabolic profiles and expressions, offers novel insights into the realm of disease biomarkers (Figure 12).^[^ ^261^ ^]^ These profiles are highly susceptible to demographic and environmental influences, making them distinct from traditional clinical analytes and prone to false identification of biomarkers.^[^ ^25^, ^247^ ^]^

**Figure 13:**
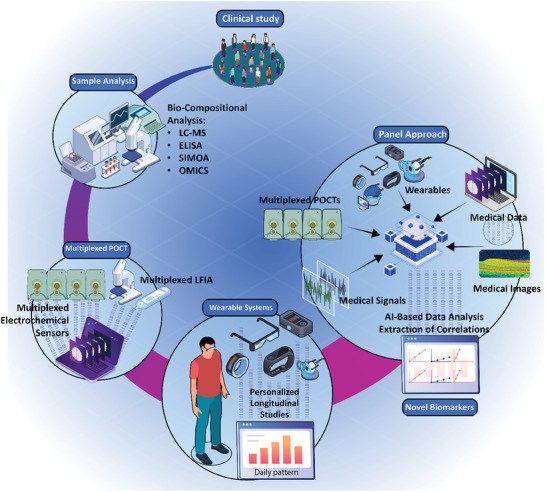
Utilization of POC systems and wearable platforms for biomarker discovery. Moving from conventional biomolecular and biocompositional analysis methods for biomarker discovery in clinical studies, such as omics approaches, LC‐MS, or ELISA, POC biomarker detection systems can be used in a high‐throughput format, remotely analyzing biosamples or medical image/data for extracting patterns and discovering new features/markers. AI methods can play a pivotal role in introducing reliable disease‐specific biomarkers by analyzing the collected data from multiple sources including wearable systems, medical data, medical images, medical signals, and multiplexed POCT systems, and then drawing on correlations and associations.

When evaluating methods for discovering diagnostic and prognostic biomarkers for diseases, it is crucial to recognize that no single method consistently outperforms others. Utilizing multiple methods is essential to enhance the chances of identifying an optimal model, considering the dataset's limitations. This can be achieved through an integration approach, which combines various biomarker discovery methods, leveraging their respective strengths and mitigating weaknesses (**Figure** **13**). Function perturbation and data perturbation strategies are employed, where data subsets are generated from the original dataset, and the results are combined to improve method stability. To maximize robustness, a novel framework integrates both data and function perturbation, utilizing stability calculations as weights when merging results from multiple methods.^[^ ^254^ ^]^ The integration of ML techniques with other methods, such as targeted metabolomics using Liquid Chromatography‐Mass Spectroscopy (LC‐MS), presents a promising interdisciplinary approach for early cancer diagnosis, offering more accurate and rapid screening tools that could extend beyond lung cancer to enhance precision oncology in complex disease understanding. This pioneering interdisciplinary method not only demonstrates significant diagnostic strength for early detection of lung tumors but also addresses challenges like data scarcity, interpretability, and standardization, making it a valuable contribution to advancing precision oncology through the use of multimodal data models.^[^ ^262^ ^]^ Figure 13 demonstrates the path for biomarker discovery from established bio‐compositional analysis methods to the use of POCTs and wearables, in particular in a multiplexed panel format.

## 5 Conclusion and Perspectives

In this paper, the most recent technological advances in the areas pertaining to wearable POC platforms and diagnostic solutions have been discussed. We showcased the current trend in the development of in vitro diagnostics and their integration with smart technologies, along with the use of digital data and AI for disease detection and operation of bioassays, as well as advances in biomarker discovery, as an important aspect of all diagnosis and prognosis tools. The growing advancements in these fields lead to the emergence of new applications emerging in the biomedical area, serving the health industry. The applications expand to sectors such as diagnostics and care, pharmaceuticals and drug discovery, and food and sports medicine. On the other hand, technological innovations in the field of POC devices and wearable platforms, not only have the potential for transforming the early diagnosis practice, but also can be vastly incorporated for discovering new disease markers, whether it be biomolecules, or medical imaging/signal features.^[^ ^271^ ^]^ This is particularly important for diseases where the medical criteria for diagnosis are not well‐defined, such as mental health conditions, including anxiety and depression. It is interesting to see how new technologies such as wearable platforms, integrated with AI, are contributing to the psychiatric realm particularly for diagnosis purposes.^[^ ^272^ ^]^ Expanding interest in this field has even attracted the attention of the community toward incorporating analytical techniques, such as electrochemical biosensing, for both biomarker discovery and diagnosis of Major Depressive Disorder (MDD).^[^ ^273^ ^]^

Beyond the diagnostic realm, the idea of integrating wearable and POC solutions with AI technologies, can also staggeringly benefit the therapeutic practice. Digital biomarkers,^[^ ^274^ ^]^ which are the digital traces of individuals' bio‐physiological conditions, acquired mostly using wearable systems, along with disease‐specific biomolecular markers, can be utilized by pharmaceutical companies for meticulously monitoring and enhancing the efficacy of drugs and therapeutic solutions. Wearable systems can provide a large amount of comprehensive precise data in response to the treatments, by analyzing the biosamples or the physiological conditions. Regardless of the strategies undertaken by the pharmaceutical companies, e.g., product‐based, or service‐oriented, the opportunity of adding “big data” analysis to the process, especially with AI tools, is a unique chance for developing cutting‐edge treatment approaches. Improving the outcomes of clinical trials by the involvement of more and more high‐end technologies and investing in Real‐World Evidence‐based (RWE) programs^[^ ^275^ ^]^ would be among the advantages of converging engineering technologies with healthcare practice.^[^ ^276^ ^]^ For biomarker discovery, in the digital pathology practice, it is important to perform multistep validation in order to ensure that the AI algorithms and the methodology have clinical usefulness. In this area, eliminating the manual processes can tremendously impact the clinical outcomes, which is a precursor to ensuring the reliability of the data collection and the engineering process through which the biomarkers are being developed.

Biomarker detection using near‐patient methods, such as wearables and POCs, and through AI‐integrated systems, as one of the overarching trends in the biomedical field, is a key factor through which clinical practitioners can practice “personalized medicine”. Via this approach, each patient will have access to advanced personalized health monitoring gadgets, in the form of wearables or POC platforms, that can be used on a regular basis, providing both patients and physicians with longitudinal health data that can help with enhanced monitoring of the individual's health conditions and effectiveness of the prescribed therapeutic approaches. It is an undeniable fact that the real‐world utility of such technologies is contingent upon rigorous monitoring for regulatory approval, and enhanced utility in the real‐world near‐patient conditions. One key aspect though, is the robustness of the POC/wearable solutions, in particular for biomolecular analysis, which is needed for accurate and reliable data classification and processing using AI/ML algorithms. In terms of the ML algorithms themselves, the enhancement of the interpretability of algorithms, which is referred to as the “black box”, will assist with the validation process, and consequently regulatory approval and smooth commercialization of products. In order to have a viable product, that can be utilized in real‐world settings, beyond the laboratory conditions, having access to a panel of biomarkers that enables multi‐marker sensing is vital. A current challenge in this area is limited funding allocations to discovery research, which is also considered a high‐risk high‐reward research. While some research institutes have realized the importance of rewarding this type of research and inviting principal investigators to utilize the clinical resources for biomarker discovery, more interdisciplinary collaborations are required for designing the clinical studies, participant recruitment, data analysis, and technology development. Augmenting the multiplexing techniques with big data processing undoubtedly creates enormous opportunities for having access to a panel of markers, which collectively can be indicators of specific diseases. It is worth noting that less invasive techniques for specimen collection and analysis, which are mainly enabled via wearable sensing, also empower remote monitoring and consequently enlargement of the clinical cohort, which increases the reliability of clinical studies and drug testing trials.

Wearable and POC systems are recognized as biomarkers detection solutions as they are assumed to offer a lower cost in comparison with centralized methods. There is also no question that the costs associated with the integration of AI‐integrated wearable/POC solutions into the early diagnosis and biomarker discovery practices is one of the key elements in the path toward market/clinic translation and is one of the factors that impose restrictions on large‐scale usability. While the cost of on‐site testing would be presumably less than overall expenses incurred by multiple laboratory or physician visits, money‐wise and timewise, expenses related to trained personnel conducting the tests and the subsequent interpretation of results, both in terms of financial and time considerations, should be taken into account. However, careful considerations should go toward monitoring the manufacturing costs of wearable/POC systems, the actual value of adding AI tools for data processing, IP protection, and shipping and handling expenses, among others. In the long run, as they become established as standard procedures, wearable systems can also assist in introducing alternative body fluids which will eventually replace the invasive blood testing methods. As discussed earlier in this text, the POC platforms should also be endowed with algorithms capable of accounting for the potential presence of bias, particularly in the context of AI‐based data processing. Many algorithms, when trained on a specific set of data lacking essential diversity, are susceptible to biases, which should be diligently examined before, during, and after analysis to avoid misleading results. Currently, transitioning these products from research laboratories to the market may encounter hurdles related to regulatory approvals and minimal investment during the Minimum Viable Product (MVP) development stage. This stage is critical for supporting the innovation of the more functional versions of a device. Nevertheless, it is encouraging to see many universities and research institutes, as well as accelerators and incubators for research‐based start‐ups, provide growing support for facilitating innovation translation.

While continual efforts are still being made for widespread utilization of POC systems and wearable platforms integrated with AI solutions, the commercialized examples, and the regulatory efforts put in place, all indicate that we are not far from witnessing such technologies incorporated into the patient's daily lives. An interdisciplinary effort would be needed not only to develop precision medicine technologies but also to translate them and ensure their reliability in patient or clinical use. With more and more advancements made in this area, the acquired data can also be used as a feedback system for enhancing the functionality and accuracy of AI systems. Altogether, such technologies will undoubtedly be considered a leap forward in the realm of diagnostics and therapeutics, providing enormous research opportunities for further characterizing the diseases, and improving the efficacy of the treatments and medications, in addition to the early‐stage disease detection and health condition monitoring.

## Conflict of Interest

The authors declare no conflict of interest.
